# Liraglutide vs Semaglutide vs Dulaglutide in Veterans With Type 2 Diabetes

**DOI:** 10.1001/jamanetworkopen.2025.37297

**Published:** 2025-10-13

**Authors:** Catherine G. Derington, Amara Sarwal, Guo Wei, Sydney E. Hartsell, Michael Throolin, Ravinder Singh, McKenna R. Nevers, Chong Zhang, Niharika Katkam, Augustine Takyi, Akhil R. Chakravartula, Poorvika Babu, Vikrant G. Deshmukh, Robert E. Boucher, Stavros G. Drakos, Tom Greene, Jincheng Shen, Srinivasan Beddhu

**Affiliations:** 1 Department of Medicine, Division of Cardiology, University of Colorado School of Medicine, Aurora; 2 Adult & Child Center for Outcomes Research & Delivery Science, University of Colorado Anschutz Medical Campus, Aurora; 3 Division of Nephrology and Hypertension, Department of Internal Medicine, University of Utah School of Medicine, Salt Lake City; 4 Cardiovascular, Renal and Metabolism Center, University of Utah School of Medicine, Salt Lake City; 5 Division of Biostatistics, Departments of Population Health Sciences, University of Utah School of Medicine, Salt Lake City; 6 Veterans Affairs Salt Lake City Health Care System, Salt Lake City, Utah; 7 Nora Eccles Harrison Cardiovascular Research & Training Institute, University of Utah, Salt Lake City; 8 Division of Epidemiology, Department of Internal Medicine, University of Utah School of Medicine, Salt Lake City; 9 Informatics, Decision-Enhancement, and Analytic Sciences Center of Innovation, Veterans Affairs Salt Lake City Health Care System, Salt Lake City, Utah; 10 Division of Cardiovascular Medicine, Department of Internal Medicine, University of Utah School of Medicine, Salt Lake City

## Abstract

**Importance:**

The head-to-head comparative effectiveness and safety of individual glucagon-like peptide-1 receptor agonists (GLP-1RAs) are not well understood.

**Objective:**

To compare risks of kidney, cardiovascular, and death outcomes among patients with type 2 diabetes initiating GLP-1RAs in the Department of Veterans Affairs (VA) health system.

**Design, Setting, and Participants:**

This comparative effectiveness study used an active-comparator, new-user target trial–emulation design with national data linked among the VA, Medicare, and US Renal Data System. Participants were GLP-1RA–naive veterans with type 2 diabetes and without end-stage kidney disease treated with metformin who started liraglutide, semaglutide, or dulaglutide between January 1, 2018, and December 31, 2021. Data were analyzed from September 2024 to June 2025.

**Exposure:**

Liraglutide, semaglutide, or dulaglutide.

**Main Outcomes and Measures:**

Kidney failure (sustained estimated glomerular filtration rate <15 mL/min/1.73 m^2^ or initiation of kidney replacement therapy), composite cardiovascular and kidney metabolic (CKM) events (kidney failure or major adverse cardiovascular events [MACE]; myocardial infarction, heart failure, or stroke/transient ischemic attack), MACE, all-cause death, and adverse gastrointestinal events (gastroparesis, intestinal obstruction, gallstones, acute cholecystitis, acute pancreatitis) were evaluated separately through March 31, 2023.

**Results:**

Of 21 790 included veterans (mean [SD] age, 63.5 [10.8] years, 19 823 [91.0%] male), 5425 (24.9%), 10 838 (49.7%), and 5527 (24.9%) initiated liraglutide, semaglutide, and dulaglutide, respectively. In weighted Cox regression models, compared with initiation of semaglutide, liraglutide initiation had similar hazards for kidney failure (hazard ratio [HR], 0.93; 95% CI, 0.60-1.44), the CKM composite outcome (HR, 0.96; 95% CI, 0.84-1.10), and MACE (HR, 0.95; 95% CI, 0.83-1.09). Results were similar with liraglutide vs dulaglutide and dulaglutide vs semaglutide comparisons. Liraglutide had significantly lower hazard of all-cause death compared with semaglutide under intent-to-treat analyses (HR, 0.83; 95% CI, 0.69-0.99), which lost significance in per-protocol models. Compared with dulaglutide, liraglutide was associated with a lower risk of all-cause mortality in both the intent-to-treat (HR, 0.69; 95% CI, 0.58-0.83) and per-protocol (HR, 0.50; 95% CI, 0.31-0.82) analyses, but compared with semaglutide, dulaglutide had higher hazard of mortality only in the per-protocol model (HR, 1.72; 95% CI, 1.20-2.47). The only observed difference for the gastrointestinal adverse events was a decreased risk for gallstones and acute cholecystitis with dulaglutide vs semaglutide (gallstones: HR, 0.72; 95% CI, 0.54-0.95; acute cholecystitis: HR, 0.62; 95% CI, 0.39-0.99).

**Conclusions and Relevance:**

In this comparative effectiveness study in veterans with diabetes, liraglutide, semaglutide, and dulaglutide initiators had similar risks for kidney and cardiovascular outcomes. Head-to-head randomized trials are needed to confirm these findings.

## Key Points

**Question:**

Are different glucagon-like peptide-1 receptor agonists associated with differences in kidney and cardiovascular outcomes among patients with type 2 diabetes?

**Findings:**

In this comparative effectiveness study of 21 790 veterans with type 2 diabetes and without end-stage kidney disease, kidney and cardiovascular outcomes were similar among liraglutide, semaglutide, and dulaglutide initiators.

**Meaning:**

These findings highlight the need for head-to-head randomized clinical trials to definitively establish potential differences among glucagon-like peptide-1 receptor agonists.

## Teaser

This comparative effectiveness study examines risks of kidney, cardiovascular, and death outcomes among patients with type 2 diabetes initiating glucagon-like peptide-1 receptor agonists in the Veterans Health Administration.

## Introduction

Among the 29.7 million US adults with diagnosed type 2 diabetes, cardiovascular disease (CVD) is the leading cause of mortality.^1^ Furthermore, 30% to 40% of patients with diabetes develop chronic kidney disease, leading to significant morbidity and health care costs.^2,3,4^ Cardiovascular-kidney-metabolic (CKM) syndrome is an emerging focus for patients with diabetes, highlighting the shared pathophysiology among diabetes, CVD, and chronic kidney disease.^5^

Among therapeutic options for diabetes, glucagon-like peptide-1 receptor agonists (GLP-1RAs) offer important advantages for patients with CKM. In pivotal randomized clinical trials, compared with placebo, GLP-1RAs decreased all-cause mortality, CVD, and kidney function decline in patients with diabetes.^6,7,8,9^ Given their effectiveness and safety, use of GLP-1RAs has grown substantially since exenatide first became available in 2005. As of 2022, more than 5.1 million patients with diabetes use GLP-1RAs.^10^ Nonetheless, cost and equitable access to GLP-1RAs remain an issue.^11^

As the GLP-1RA market expands and generic GLP-1RAs become available starting in 2025, understanding intraclass differences on clinical outcomes is essential. Beyond glycemic control, liraglutide, semaglutide, and dulaglutide are all approved by the US Food and Drug Administration (FDA) to prevent CVD outcomes in patients with diabetes.^6,7,8,9^ Although all 3 drugs have shown beneficial effects on early markers of kidney damage (ie, decreased albuminuria or prevented declines in estimated glomerular filtration rate [eGFR]), evidence is limited on their effects as a class on more advanced kidney outcomes (eg, progression to end-stage kidney disease). Only semaglutide is approved in the US by the FDA for slowing chronic kidney disease progression in patients with diabetes and chronic kidney disease.^6,7,9,12^ Furthermore, to our knowledge, no head-to-head trial has directly compared effects of individual GLP-1RAs on clinical events. One indirect meta-analysis of 7 trials including 56 004 participants suggested that semaglutide was associated with lower rates of CVD events and CVD death,^13^ and only 1 cohort study reported similar effectiveness between liraglutide and dulaglutide for CVD and kidney outcomes.^14^

To address this gap in the comparative effectiveness literature and to inform the next era of GLP-1RA use in the US, we adopted the active-comparator, new-user design to study kidney, cardiovascular, and mortality outcomes among patients with type 2 diabetes initiating the 3 most common GLP-1RAs used in the Department of Veterans Affairs (VA) health system—liraglutide, semaglutide, and dulaglutide—between 2018 and 2021.

## Methods

This comparative effectiveness study was approved by the University of Utah institutional review board with a waiver of informed consent due to the use of deidentified retrospective data. This study followed the Strengthening the Reporting of Observational Studies in Epidemiology (STROBE) reporting guideline.

### Data Sources and Study Oversight

This retrospective comparative effectiveness study used national, patient-level data from the VA. These data include information since October 1, 1999, from clinical encounters, pharmacy dispenses, and administrative claims for 172 VA medical centers and 1138 VA outpatient clinics in the US.^15^ A detailed description of the VA data files used in this study are provided in the eMethods in Supplement 1.^16,17^

### Study Design and Population

We used an active-comparator, new-user cohort study design to emulate a target trial comparing effectiveness and safety outcomes among patients with type 2 diabetes initiating liraglutide, semaglutide, or dulaglutide.^18,19,20^ The generation of the study cohort has been previously described.^21^ The first dispense date of a GLP-1RA during the study period denoted the index date and start of follow-up for each veteran. From 2 462 623 veterans with an all-time history of type 2 diabetes diagnosis using *International Classification of Diseases, Ninth Revision (ICD-9) *and* International Statistical Classification of Diseases and Related Health Problems, Tenth Revision (ICD-10) *codes who were dispensed a prescription for a GLP-1RA, sodium-glucose cotransporter-2 inhibitor (SGLT2I), or insulin glargine between January 1, 2018, and December 31, 2021, we further excluded 307 323 veterans (12.5%) not using metformin at baseline; 137 028 veterans (6.4%) who were prevalent users or coexposed to 2 GLP-1RAs, SGLT2I, or insulin glargine on the index date; 139 260 veterans (6.9%) who did not initiate a GLP-1RA; 286 veterans (<0.1%) who initiated exenatide, albiglutide, tirzepatide, or lixisenatide; and 69 veterans (<0.1%) with a baseline history of end-stage kidney disease or kidney transplant. The final study cohort included 21 790 new users of liraglutide, semaglutide, or dulaglutide (eFigure 1 in Supplement 1).

### Baseline Variables

Baseline information was obtained from data available on or before the index date (eTable 1 in Supplement 1). Among other demographics, data on race and ethnicity were self-reported based on enrollment files, further categorized as Hispanic, non-Hispanic Black, non-Hispanic White, and non-Hispanic other (including multiracial, non-Hispanic American Indian or Alaska Native, Non-Hispanic Asian or Pacific Islander, and missing). We included variables on race and ethnicity because there are documented racial and ethnic disparities in GLP-1RA access in the VA.^22,23^ The most recent physical and laboratory measurements in the 1 year before the index date were selected for body mass index and eGFR (calculated from measured serum creatinine using the race-neutral Chronic Kidney Disease Epidemiology Collaboration creatinine equation).^24^ We calculated the mean from the 3 most recent outpatient systolic and diastolic blood pressure measurements in the 1 year prior to the index date, separately. Information about diagnoses was obtained from all available claims before the index date to identify prevalent conditions and diabetes history. Pharmacy fill data from 6 months prior to the index date defined prevalent use of antihyperglycemics, antihypertensives, and statins. Follow-up medication histories were constructed for each patient using dispense dates, quantity dispensed, and days’ supply, allowing assessment of adherence, dose, and therapeutic switches.

### Outcomes

Follow-up was until loss to follow-up, death, or March 31, 2023. Complete definitions of outcomes are provided in eTable 2 in Supplement 1. All nondeath events were ascertained from diagnosis codes attached to VA and non-VA care encounters, the VA-linked US Renal Data System, and measurements available in the Corporate Data Warehouse.

We separately examined 3 effectiveness outcomes: kidney failure, CKM composite, and CVD events (ie, major adverse cardiovascular events [MACE]). Kidney failure was defined as sustained eGFR less than 15 mL/min/1.73 m^2^ or development of end-stage kidney disease (initiation of kidney replacement therapy or preemptive kidney transplant identified through the US Renal Data System until December 31, 2021, or VA *ICD-10* codes until the administrative censor date). The CKM composite outcome was a composite of the kidney failure outcome or MACE. The MACE outcome was a composite of hospitalizations for myocardial infarction, heart failure, or stroke or transient ischemic attack. All-cause death was ascertained using VA Death Ascertainment File. We additionally examined each effectiveness outcome as a composite with death, separately. We also separately assessed gastrointestinal adverse events (gastroparesis, intestinal obstruction, gallstones, acute cholecystitis, and acute pancreatitis).

### Statistical Analysis

To account for between-group differences in baseline characteristics, we constructed a propensity score using multinomial logistic regression, which estimated the probability of initiating each medication based on 56 baseline variables (ie, index year as a categorical variable, sociodemographics, laboratory and physical measurements, disease history and co-occurring medical conditions, concomitant medication use) (eFigure 2 in Supplement 1). Missing values (eTable 3 in Supplement 1) were imputed using K-nearest neighbors imputation, except for urine albumin-to-creatinine ratio, which was coded as categorical or missing, given its high missing rate. Inverse probability of treatment weighting (IPTW) using the propensity scores was applied, truncating at the 1st and 99th percentiles for stability. Baseline characteristics were compared across treatment groups before and after weighting, with balance plots representing the maximum standardized mean difference (SMD) of the 3 groups as well as SMD for the 3 treatment comparisons. An SMD between −0.1 and 0.1 indicates that the groups are well-balanced and appropriate for causal interpretation in the absence of unmeasured confounding.^25^

We conducted pairwise comparisons to characterize the intention-to-treat (ITT) estimand: the association between initiating liraglutide, semaglutide, or dulaglutide on the outcomes of interest, irrespective of adherence or treatment switches during follow-up. Because liraglutide is the first GLP-1RA to go off-patent, and semaglutide is the first GLP-1RA to be approved by the FDA to prevent kidney decline in patients with chronic kidney disease, we assigned our primary treatment comparison of interest as liraglutide vs semaglutide. Weighted and unweighted event rates were calculated. IPTW-weighted cumulative incidence functions were plotted to visually compare the treatment-specific outcomes, including the competing risk of death, with IPTW-weighted Cox regression estimating the hazard ratios (HRs) corresponding to each treatment comparison. Absolute risk, absolute risk differences, and risk ratios at 3 years were generated from the cumulative incidence functions with 200 bootstraps to generate the 95% CIs. Preliminary analyses did not detect any violations of the proportional hazards assumption (eMethods in Supplement 1), but we included index year as a variable in the propensity score estimation, given the evolving use patterns and supply constraints of GLP-1RAs during the study period. Robust standard errors were used to generate 95% CIs. Finally, as a sensitivity analysis, we repeated the primary analysis by excluding index year in the propensity score model.

We conducted subgroup analyses by refitting the propensity score model within each subgroup and applying IPTW Cox regression to estimate HRs comparing the treatments, with Wald tests computing treatment-by-subgroup interaction *P* values. A post hoc per-protocol (PP) analysis was also conducted to ascertain the pairwise associations between initiating and continuously adhering to the index treatment and the outcomes of interest across 3-month follow-up periods (eMethods in Supplement 1).^26,27,28,29^ Finally, because the cardiometabolic benefits of GLP-1RAs are partly due to weight loss,^30^ we also evaluated mean and percentage weight change (eMethods in Supplement 1).

*P* values were 2-sided, and statistical significance was set at α = .05. All analyses were completed within the VA Informatics and Computing Infrastructure using R software version 4.3.1 (R Project for Statistical Computing). Data were analyzed from September 2024 to June 2025.

## Results

### Population Characteristics

Of 21 790 veterans (mean [SD] age, 63.5 [10.8] years, 19 823 [91.0%] male) included, 5527 (25.4%) initiated liraglutide, 10 838 (49.7%) initiated semaglutide, and 5425 (24.9%) initiated dulaglutide (Table 1). Apart from index year, there were minor differences in baseline characteristics before weighting. IPTW achieved covariate balance among groups, including index year (eFigure 3 in Supplement 1).

**Table 1.** Baseline Characteristics of the Study Population

Characteristic	Unweighted			Inverse probability weighted, %		
	Liraglutide (n = 5425)	Semaglutide (n = 10 838)	Dulaglutide (n = 5527)	Liraglutide	Semaglutide	Dulaglutide
Index year						
2018	2684 (49.5)	445 (4.1)	1816 (32.9)	23.4	23.2	24.2
2019	1544 (28.5)	2197 (20.3)	1912 (34.6)	27.1	25.5	27.1
2020	769 (14.2)	2419 (22.3)	1624 (29.4)	22.5	22.1	23.4
2021	428 (7.9)	5777 (53.3)	175 (3.2)	27.0	29.3	25.3
Demographics						
Age, mean (SD), y	62.9 (10.7)	63.6 (11.0)	63.8 (10.6)	63.3 (10.8)	63.7 (10.7)	64.2 (10.8)
Sex						
Female	523 (9.6)	1022 (9.4)	422 (7.6)	9.2	9.0	8.4
Male	4902 (90.4)	9816 (90.6)	5105 (92.4)	90.8	91.0	91.6
Race and ethnicity						
Hispanic	700 (12.9)	1052 (9.7)	470.0 (8.5)	9.4	9.7	10.4
Non-Hispanic Black	742 (13.7)	1767 (16.3)	958.0 (17.3)	16.3	15.8	15.4
Non-Hispanic White	3784 (69.8)	7543 (69.6)	3876.0 (70.1)	70.3	70.3	69.6
Non-Hispanic other^a^	199 (3.7)	476 (4.4)	223.0 (4.0)	4.0	4.2	4.6
Completed high school (individual)	2130 (39.3)	4430 (40.9)	2210 (40.0)	40.7	40.5	39.5
Completed high school (household)	3777 (69.6)	7735 (71.4)	3860 (69.8)	71.5	69.9	69.7
Household income <$50 000/y	2470 (45.5)	4957 (45.7)	2540 (46.0)	45.5	45.1	45.3
Rural or highly rural residence	1877 (34.6)	3773 (34.8)	2206 (39.9)	35.2	35.3	34.5
Clinical characteristics						
Duration of diabetes, mean (SD), y	8.1 (5.5)	8.5 (5.8)	8.5 (5.6)	8.5 (5.7)	8.5 (5.6)	8.6 (5.6)
Any cardiovascular disease	2745 (50.6)	4945 (45.6)	2443 (44.2)	46.2	47.6	50.3
Coronary artery disease or revascularization	2152 (39.7)	3605 (33.3)	1761 (31.9)	34.8	35.7	36.1
Heart failure	769 (14.2)	1390 (12.8)	650 (11.8)	12.3	13.3	13.2
Cerebrovascular disease	855 (15.8)	1617 (14.9)	761 (13.8)	14.6	15.7	18.4
Cardiomyopathy	773 (14.2)	1544 (14.2)	757 (13.7)	13.6	14.5	15.3
Peripheral vascular disease	1224 (22.6)	2260 (20.9)	1050 (19.0)	20.3	21.8	21.0
Hypertension	4927 (90.8)	9821 (90.6)	5031 (91.0)	90.9	91.0	91.9
Atrial fibrillation/flutter	621 (11.4)	1324 (12.2)	575 (10.4)	11.7	11.8	12.2
Chronic lung disease	1948 (35.9)	3728 (34.4)	1745 (31.6)	34.7	34.1	32.8
Sleep apnea	2951 (54.4)	5887 (54.3)	2641 (47.8)	53.1	52.7	50.4
Cancer	736 (13.6)	1594 (14.7)	738 (13.4)	13.8	14.2	15.0
HIV/AIDS	26 (0.5)	70 (0.6)	27 (0.5)	0.5	0.7	0.6
Traumatic brain injury	146 (2.7)	304 (2.8)	120 (2.2)	2.9	2.6	2.7
Posttraumatic stress disorder	1616 (29.8)	3302 (30.5)	1579 (28.6)	28.9	29.5	28.6
Depression	2679 (49.4)	5228 (48.2)	2553 (46.2)	47.4	47.7	47.1
Anxiety	1188 (21.9)	2685 (24.8)	1056 (19.1)	22.5	22.4	21.8
Tobacco use disorder	1477 (27.2)	3134 (28.9)	1545 (28.0)	27.5	28.6	29.4
Alcohol use disorder	675 (12.4)	1465 (13.5)	634 (11.5)	12.1	12.3	13.1
Diabetic retinopathy	1494 (27.5)	2489 (23.0)	1493 (27.0)	28.8	28.1	30.5
Diabetic neuropathy	1979 (36.5)	3972 (36.6)	2058 (37.2)	37.0	38.2	37.6
Serious hypoglycemia	174 (3.2)	320 (3.0)	175 (3.2)	3.3	3.1	3.2
Gastrointestinal conditions	525 (9.7)	1095 (10.1)	468 (8.5)	9.7	9.7	9.4
Physical and laboratory measurements, mean (SD)						
Blood pressure, mm Hg						
Systolic	134 (14)	136 (17)	134 (15)	135 (16)	135 (16)	135 (16)
Diastolic	77 (9)	78 (9)	78 (9)	78 (9)	78 (9)	77 (9)
Blood pressure day count	7 (8)	5 (7)	6 (6)	6 (7)	6 (8)	6 (7)
BMI	37 (7)	35 (7)	35 (7)	36 (7)	35 (7)	35 (7)
Hemoglobin A_1c_, %						
Mean (SD)	8.5 (1.7)	8.9 (1.8)	8.9 (1.6)	8.8 (1.9)	8.8 (1.6)	8.9 (1.6)
<7	926 (17.1)	1205 (11.1)	414 (7.5)	16.1	10.3	9.6
7 to <8.5	1853 (34.2)	3617 (33.4)	1901 (34.4)	28.9	35.6	32.9
8.5 to <10	1708 (31.5)	3411 (31.5)	1969 (35.6)	31.6	32.8	34.8
≥10	938 (17.3)	2605 (24.0)	1243 (22.5)	23.5	21.3	22.8
eGFR, mL/min/1.73 m^2^						
Mean (SD)	82 (20)	80 (20)	80 (20)	81 (20)	80 (20)	81 (20)
≥60	4543 (83.7)	8882 (82.0)	4534 (82.0)	82.3	81.0	81.3
45 to <60	650 (12.0)	1389 (12.8)	698 (12.6)	12.9	13.7	12.5
30 to <45	212 (3.9)	515 (4.8)	277 (5.0)	4.3	4.9	6.0
<30	20 (0.4)	52 (0.5)	18 (0.3)	0.5	0.5	0.3
Urine albumin-to-creatinine ratio, mg/g						
<30	1907 (35.2)	3898 (36.0)	1755 (31.8)	35.2	35.3	34.2
30 to <100	622 (11.5)	1452 (13.4)	589 (10.7)	12.3	12.7	13.5
100 to <300	292 (5.4)	702 (6.5)	275 (5.0)	5.2	5.8	6.5
≥300	217 (4.0)	463 (4.3)	209 (3.8)	4.4	4.4	4.5
Not available	2387 (44.0)	4323 (39.9)	2699 (48.8)	42.9	41.8	41.3
Cholesterol, mg/dL	156 (42)	157 (43)	156 (42)	157 (43)	155 (43)	155 (42)
Total						
HDL	39 (11)	39 (10)	39 (11)	39 (11)	39 (10)	39 (11)
LDL	82 (33)	83 (35)	83 (34)	84 (34)	82 (35)	82 (33)
Triglycerides	203 (156)	203 (168)	201 (145)	204 (168)	200 (167)	202 (153)
Medications						
DPP-IV inhibitor	1186 (21.9)	3217 (29.7)	1560 (28.2)	26.6	26.6	27.4
Sulfonylurea	2700 (49.8)	5656 (52.2)	3385 (61.2)	51.3	53.4	57.4
Thiazolidinedione	510 (9.4)	998 (9.2)	677 (12.2)	10.2	9.9	9.8
Meglitinide	30 (0.6)	16 (0.1)	16 (0.3)	0.4	0.2	0.3
Insulin	1316 (24.3)	2308 (21.3)	1104 (20.0)	23.9	21.7	22.2
Alpha glucosidase	96 (1.8)	154 (1.4)	68 (1.2)	1.3	1.5	1.6
Diuretic	1860 (34.3)	3439 (31.7)	1794 (32.5)	31.2	32.5	33.2
ACEI/ARB	3852 (71.0)	7416 (68.4)	3963 (71.7)	70.1	70.3	70.5
Centrally acting agent	76 (1.4)	136 (1.3)	72 (1.3)	1.3	1.5	1.3
α-1 Blocker	1341 (24.7)	2723 (25.1)	1367 (24.7)	23.5	24.4	24.7
β-Blocker	2267 (41.8)	4016 (37.1)	2046 (37.0)	38.1	38.7	39.9
Calcium channel blocker	1500 (27.6)	2983 (27.5)	1539 (27.8)	27.1	28.2	29.5
Vasodilator	93 (1.7)	184 (1.7)	112 (2.0)	1.8	1.9	2.0
Statin	4368 (80.5)	8726 (80.5)	4466 (80.8)	79.7	80.7	81.1

Abbreviations: ACEI, angiotensin converting enzyme inhibitor; ARB, angiotensin receptor blocker; ASMD, absolute standardized mean difference; BMI, body mass index (calculated as weight in kilograms divided by height in meters squared); DPP-IV, dipeptidyl peptidase IV; eGFR, estimated glomerular filtration rate; HDL, high-density lipoprotein; LDL, low-density lipoprotein.

SI conversion factors: To convert hemoglobin A_1c_ to proportion of total hemoglobin, multiply by 0.01; cholesterol to millimoles per liter, multiply by 0.0259; triglycerides to millimoles per liter, multiply by 0.0113.

^a^ Includes American Indian or Alaskan Native, Asian American, Native Hawaiian or Other Pacific Islander, multiracial, and missing.

### Clinical Effectiveness Outcomes

#### ITT Models

**Figure 1.**
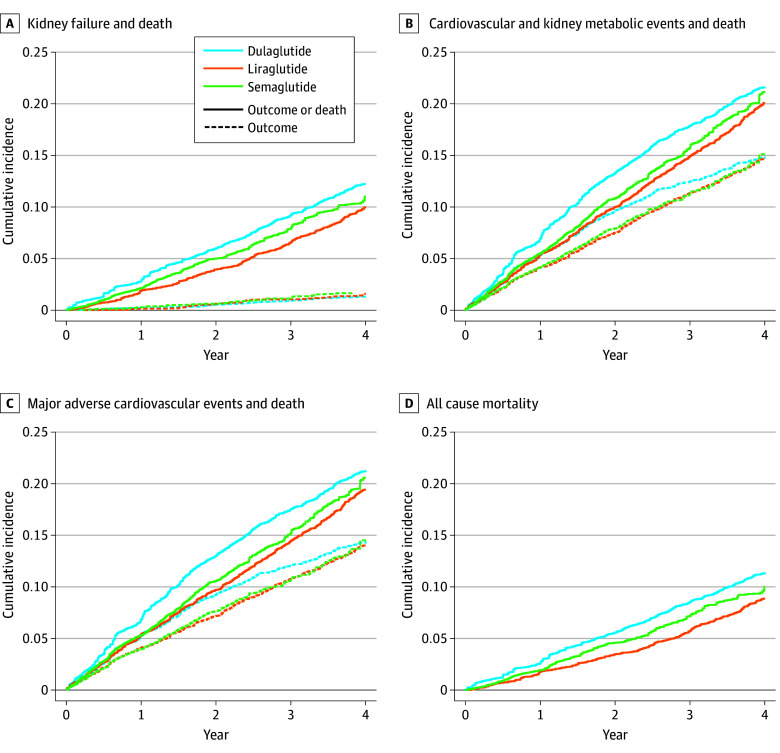
Weighted Cumulative Incidence Curves for the Clinical Outcomes Comparing Liraglutide, Semaglutide, and Dulaglutide Kidney failure includes sustained chronic kidney disease stage V (estimated glomerular filtration rate <15 mL/min/1.73 m^2^ over 60 consecutive days) or workup for transplant. Cardiovascular and kidney metabolic events is a composite of the kidney failure and major adverse cardiac events outcomes. Major adverse cardiac events include myocardial infarction, heart failure, and stroke or transient ischemic attack. Numbers at risk are not included because the figure is generated from a weighted analysis on a pseudopopulation.

The median follow-up time ranged from 2.74-3.01 years across outcomes (eTable 4 in Supplement 1). The unweighted and weighted event rates for each effectiveness outcome are shown in eTable 5 in Supplement 1. Weighted cumulative incidences of kidney failure, the CKM composite, and MACE were similar across the 3 treatment groups; however, liraglutide had the lowest incidence of all-cause death (Figure 1).

**Figure 2.**
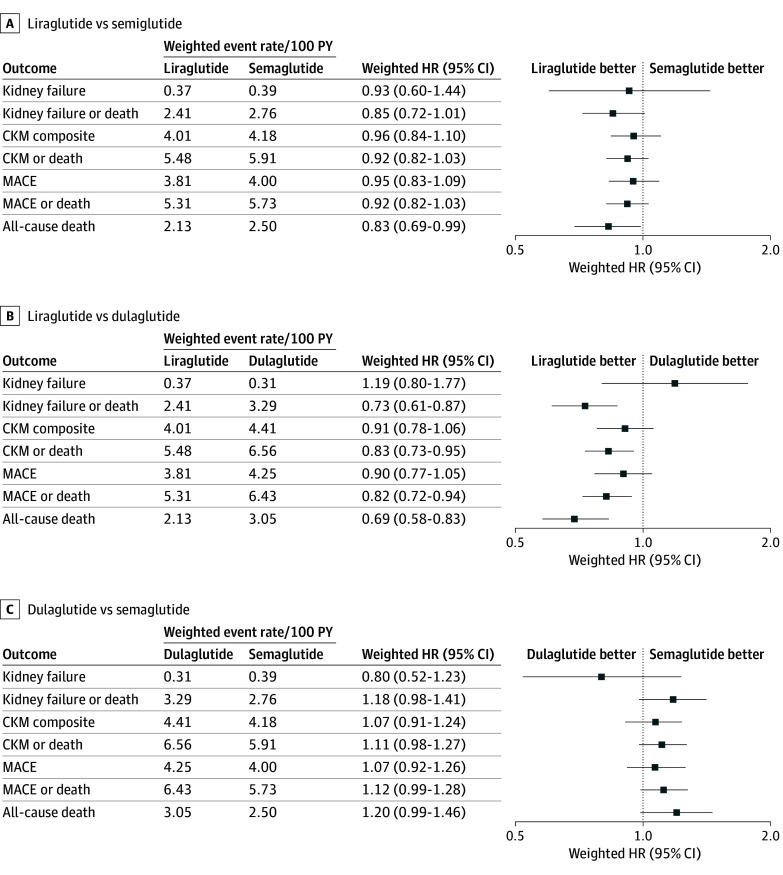
Weighted Hazard Ratios (HRs) of the Clinical Outcomes Comparing Liraglutide, Semaglutide, and Dulaglutide Kidney failure includes sustained chronic kidney disease stage V (estimated glomerular filtration rate <15 mL/min/1.73 m^2^ over 60 consecutive days) or workup for transplant. Cardiokidney metabolic composite events (CKM) composite is a composite of the kidney failure and major adverse cardiovascular events (MACE) outcomes. MACE includes myocardial infarction, heart failure, and stroke/transient ischemic attack.

In separate, weighted Cox regression models, compared with initiation of semaglutide, liraglutide initiation had similar hazards for kidney failure (HR, 0.93; 95% CI, 0.60-1.44), the CKM composite outcome (HR, 0.96; 95% CI, 0.84-1.10), and MACE (HR, 0.95; 95% CI, 0.83-1.09) but significantly lower hazard of all-cause death (HR, 0.83; 95% CI, 0.69 to 0.99) (Figure 2A). Results were similar with the liraglutide vs dulaglutide comparison (Figure 2B). There were no differences in the risks of kidney failure, the CKM composite event, MACE, or all-cause death between dulaglutide vs semaglutide initiators (Figure 2C). Results remained consistent in models assessing 3-year risk ratios (eTable 6 in Supplement 1) and removing index year from the propensity score (eTable 7 in Supplement 1).

#### PP Models

Among those alive at month 12, the unadjusted proportion of patients who were still taking their treatment was 3242 of 5326 veterans (60.9%) for liraglutide, 7071 of 10 527 veterans (67.2%) for semaglutide, and 3880 of 5382 veterans (72.1%) for dulaglutide (eTable 8 in Supplement 1). The proportions switching to another GLP-1RA within the first 12 months was 449 of 5326 veterans (8.4%), 136 of 10 527 veterans (1.3%), and 453 of 5382 veterans (8.4%) for liraglutide, semaglutide, and dulaglutide, respectively. When defining protocol adherence as at least 45 days vs at least 1 day within each 90-day follow-up period, the primary reasons for censoring were protocol violation (ie, treatment discontinuation) or loss to follow-up due to administrative censoring from the end of the study period (eTable 9 and eTable 10 in Supplement 1). Event rates were generally lower with protocol adherence defined as at least 45 days vs at least 1 day (eTable 11 in Supplement 1).

As in the ITT analysis, PP analyses demonstrated similar hazards of kidney failure, the CKM composite outcome, and MACE with liraglutide vs semaglutide (Table 2); however, the significantly lower hazard of death observed in the ITT analysis lost statistical significance in the PP analysis. Dulaglutide had similar hazards for kidney failure, CKM composite, and MACE compared to liraglutide or semaglutide, but dulaglutide had higher hazard of mortality compared with semaglutide (HR, 1.72; 95% CI, 1.20-2.47). As in the ITT analysis, compared with dulaglutide, liraglutide was associated with a lower hazard of all-cause death in the PP analysis (HR, 0.50; 95% CI, 0.31-0.82).

**Table 2.** Comparing the Weighted ITT and PP HRs for the Effectiveness Outcomes Among Veterans Initiating the Study Drugs

Outcome	HR (95% CI)								
	Liraglutide vs semaglutide			Liraglutide vs dulaglutide			Dulaglutide vs semaglutide		
	ITT	PP (≥45 d)^a^	PP (≥1 d)^b^	ITT	PP (≥45 d)^a^	PP (≥1 d)^b^	ITT	PP (≥45 d)^a^	PP (≥1 d)^b^
Kidney failure	0.93 (0.60-1.44)	0.66 (0.25-1.76)	0.89 (0.38-2.09)	1.19 (0.80-1.77)	1.07 (0.44-2.61)	1.13 (0.48-2.64)	0.80 (0.52-1.23)	0.60 (0.30-1.18)	0.78 (0.41-1.47)
Kidney failure or death	0.85 (0.72-1.01)	0.90 (0.61-1.31)	0.89 (0.66-1.21)	0.73 (0.61-0.87)	0.54 (0.34-0.84)	0.62 (0.43-0.91)	1.18 (0.98-1.41)	1.60 (1.14-2.25)	1.38 (1.02-1.88)
CKM composite	0.96 (0.84-1.10)	1.04 (0.84-1.29)	1.09 (0.89-1.33)	0.91 (0.78-1.06)	0.82 (0.63-1.06)	0.81 (0.63-1.04)	1.07 (0.91-1.24)	1.26 (1.01-1.58)	1.30 (1.05-1.62)
CKM composite or death	0.92 (0.82-1.03)	1.03 (0.84-1.25)	1.05 (0.88-1.25)	0.83 (0.73-0.95)	0.73 (0.57-0.93)	0.75 (0.60-0.93)	1.11 (0.98-1.27)	1.39 (1.13-1.71)	1.38 (1.14-1.67)
MACE	0.95 (0.83-1.09)	1.03 (0.83-1.28)	1.08 (0.89-1.33)	0.90 (0.77-1.05)	0.81 (0.62-1.05)	0.80 (0.62-1.03)	1.07 (0.92-1.26)	1.27 (1.01-1.60)	1.32 (1.05-1.65)
MACE or death	0.92 (0.82-1.03)	1.02 (0.83-1.24)	1.05 (0.88-1.25)	0.82 (0.72-0.94)	0.72 (0.56-0.92)	0.74 (0.59-0.92)	1.12 (0.99-1.28)	1.41 (1.14-1.73)	1.39 (1.15-1.69)
All-cause death	0.83 (0.69-0.99)	0.89 (0.60-1.33)	0.84 (0.61-1.16)	0.69 (0.58-0.83)	0.50 (0.31-0.82)	0.58 (0.39-0.86)	1.20 (0.99-1.46)	1.72 (1.20-2.47)	1.40 (1.00-1.95)

Abbreviations: CKM, cardiokidney metabolic; HR, hazard ratio; ITT, intention-to-treat; MACE, major adverse cardiovascular event; PP, per-protocol.

^a^ Defined as at least 45 days of adherence.

^b^ Defined as at least 1 day of adherence.

### Clinical Safety Outcomes

Semaglutide initiators had the highest unweighted incidence rates for all gastrointestinal adverse outcomes (eTable 12 in Supplement 1). However, in weighted Cox regression, the risks for each gastrointestinal adverse event were similar in both ITT (eTable 12 in Supplement 1) and PP analyses (eTable 13 in Supplement 1), except for a decreased risk for gallstones (HR, 0.72; 95% CI, 0.54-0.95) and acute cholecystitis (HR, 0.62; 95% CI, 0.39-0.99) with dulaglutide vs semaglutide.

### Secondary and Subgroup Analyses

Among those with nonmissing baseline weight (20 404 veterans [93.6%]), the estimated mean change in weight at month 24 was −9.7 (95% CI, −10.5 to −8.8) pounds with initial treatment with liraglutide, −12.2 (95% CI, −12.9 to −11.5) pounds for initial treatment with semaglutide, and −9.8 (95% CI, −10.6 to −8.9) pounds for initial treatment with dulaglutide (liraglutide vs dulaglutide: *P* = .86; liraglutide vs semaglutide: *P* < .001; dulaglutide vs semaglutide: *P* < .001) (eFigure 4 in Supplement 1). Among 30 subgroups of interest, the risks for each outcome were similar among treatment groups (eFigure 5 in Supplement 1). Statistically significant interaction *P* values should be cautiously interpreted, given the numbers of treatment groups, outcomes, and subgroups without multiple-comparison adjustment.

## Discussion

This active-comparator, new-user comparative effectiveness study has 2 main findings regarding the intraclass comparative effectiveness and safety among liraglutide, semaglutide, and dulaglutide. The first is that liraglutide, semaglutide, and dulaglutide were associated with comparable effectiveness for kidney and cardiovascular outcomes. While liraglutide and semaglutide were associated with similar risks of all-cause death, PP analyses suggested that liraglutide was associated with a lower risk of all-cause death compared with dulaglutide. Furthermore, these agents also appear to be equivalently safe with rare incidences of gastrointestinal adverse events, with a small exception that semaglutide was associated with a greater risk of gallstones and acute cholecystitis relative to dulaglutide. These findings are hypothesis-generating for a future randomized clinical trial to examine noninferiority of generic liraglutide vs semaglutide for kidney end points, as semaglutide is currently the only GLP-1RA approved by the FDA for slowing chronic kidney disease progression among patients with type 2 diabetes.

GLP-1RAs mimic endogenous mechanisms of human GLP-1^31,32^ and reduce the risk of kidney and CVD events by reducing inflammation and fibrosis, stabilizing the endothelium via decreased low-density lipoprotein cholesterol and increased nitric oxide, and promoting natriuresis.^6,31,33^ Exendin-4-based GLP-1RAs (ie, exenatide, lixisenatide), have lower receptor binding affinity compared with human analogs (ie, liraglutide, semaglutide, dulaglutide), which also reversibly bind albumin and extend their half-life.^32,34^ Semaglutide has the highest binding affinity and dulaglutide has the longest half-life, supporting their once-weekly administration and more consistent receptor activation compared with daily-administered liraglutide.^32,34,35,36^ Our findings call into question whether these differences result in clinically relevant effects. However, the most potent incretin receptor agonist, tirzepatide, has demonstrated superior weight-loss efficacy, hemoglobin A_1c_ lowering, and tolerability compared with other GLP1-RAs,^37,38,39^ likely due to its dual activity at gastric inhibitory polypeptide and GLP-1 receptors. As tirzepatide use increases, future comparative studies will be needed to evaluate its long-term effectiveness and safety relative to other incretin-based therapies.

The main findings of this study were that liraglutide and semaglutide were associated with similar risks for kidney and cardiovascular outcomes, and these findings consistent in ITT, PP, subgroup, and sensitivity analyses. Notably, event rates for the effectiveness outcomes were lower when protocol adherence for each 90-day follow-up period was defined as at least 45 days than at least 1 day, suggesting biological plausibility of these observations. Our findings also add important context to the use of GLP-1RAs in a large national health system. GLP-1RAs are expensive agents—out-of-pocket costs can exceed $1000 for 1 prescription fill^40^—and their use for weight loss and other off-label indications has caused constraints on drug supply since 2022.^41^ Patients may need to switch among individual GLP-1RAs due to effectiveness, tolerability, or changes in insurance formularies or copays, which was observed to some degree in our study. Furthermore, there was evidence of greater preference to initiate semaglutide vs liraglutide in later years of our study, likely due to formulary changes. Indeed, index year showed the highest imbalance between liraglutide and semaglutide initiation, although this imbalance was adequately attenuated with IPTW. In total, the primary findings should reassure clinicians, patients, and health systems all 3 agents are relatively similar to one another with respect to preventing nonterminal kidney and cardiovascular events, should an intraclass switch be needed based on tolerability, cost, or access.

Regarding safety, we observed a low incidence of gastrointestinal adverse events overall. The observed increased risk of gallstones with semaglutide compared with dulaglutide may be due to the former’s higher receptor affinity and associated rapid weight loss, which is independently associated with gallstones and has been observed in other clinical and observational studies of semaglutide.^38,39,40,41^ Veterans in all treatment groups of this study had similar weight loss at 24 months, with small absolute differences (less than 3 pounds) among the groups; given that these medications were dosed to treat diabetes, not weight loss, additional studies are needed to ascertain the relationship between specific GLP1-RAs, dose, weight loss, and cardiometabolic events.

### Strengths and Limitations

Strengths of this study include the active-comparator, new-user design, which minimizes indication and prevalent user biases. IPTW effectively balanced baseline characteristics to allow inferential assessment. The large sample size from clinical settings also allowed generalizability of the findings to routine clinical practice. The datasets include robust clinical information, notably pharmacy dispenses and kidney outcomes with linkage to Medicare and the US Renal Data System.

Regarding limitations, unmeasured confounding is a risk, given the observational design, although the active comparator design minimizes this potential by improving exchangeability among the groups.^19,20^ The short follow-up time may have precluded capture of long-term effects. For the primary comparison of interest (liraglutide vs semaglutide), the 95% CI extended below 0.85, indicating the potential for a risk reduction of 15% or more, while typically excluding the possibility of risk increases exceeding 15%. Accordingly, the uncertainty is asymmetric, favoring potential benefit (or no effect) over harm with one agent compared to the other. Although we detected no violations in the proportional hazards assumption, we did note smaller widths of the 95% CIs when index date was excluded from the propensity score model, suggesting that temporal prescribing patterns and supply disruptions could plausibly contribute to some of the observed results. While some subgroup *P* values for interaction were statistically significant (race and ethnicity), given the number of subgroups tested (30) across 3 treatment comparisons and 4 outcomes, some significant *P* values may reflect random chance, particularly without testing for multiple comparisons. The study population was predominantly older White men; hence, the generalizability of these results to diverse groups (eg, younger individuals, women, and members of racial or ethnic minority groups) needs to be further studied.

## Conclusions

The findings of this comparative effectiveness study provide important clinical evidence comparing associations of liraglutide, semaglutide, and dulaglutide with kidney, cardiovascular, and mortality outcomes, as well as gastrointestinal safety outcomes. All 3 agents demonstrated comparable effectiveness for kidney and cardiovascular outcomes. While liraglutide and semaglutide were equivalent for mortality, liraglutide was associated with lower mortality risk compared with dulaglutide, which requires further study. These findings support the need for a future head-to-head randomized clinical trial comparing liraglutide vs semaglutide, particularly given that the GLP-1RAs will lose patent exclusivity and become more widely accessible.

## Notes

Supplement 1.
eMethods.
**eTable 1.** Definitions of baseline variables **eTable 2.** Definitions of study outcomes **eTable 3.** Missingness of baseline characteristics overall and by treatment group **eTable 4.** Median follow-up time in the overall cohort by outcome **eTable 5.** Incidence rates for the effectiveness outcomes among veterans initiating the study drugs **eTable 6.** Absolute risk, absolute risk differences, and risk ratios at 3 years between the treatment groups for each outcome of interest **eTable 7.** Sensitivity analysis removing index year from the propensity score model **eTable 8.** 12-Month treatment discontinuation and switch rates among study cohort with 1 complete year of follow-up data **eTable 9.** Reasons for censoring at each 3-month interval in the per-protocol analysis (protocol A, adhering for at least 45 days during each interval) **eTable 10.** Reasons for censoring at each 3-month interval in the per-protocol analysis (protocol B, adhering for at least 1 day during each interval) **eTable 11.** Weighted per-protocol event rates for the effectiveness and safety outcomes among veterans initiating the study drugs **eTable 12.** Intent-to-treat gastrointestinal adverse events among veterans initiating the study drugs **eTable 13.** Comparing the weighted intent-to-treat and per-protocol hazard ratios for the effectiveness outcomes among veterans initiating the study drugs **eFigure 1.** Flowchart of veteran selection **eFigure 2.** Histograms of the propensity score by treatment group **eFigure 3.** Balance of baseline characteristics before and after weighting **eFigure 4.** Weight change at 24 months among Veterans initiating liraglutide, semaglutide, and dulaglutide **eFigure 5.** Subgroup results
eReferences.

Supplement 2.
Data Sharing Statement

## References

[1] Low Wang CC, Hess CN, Hiatt WR, Goldfine AB. Clinical Update: cardiovascular disease in diabetes mellitus: atherosclerotic cardiovascular disease and heart failure in type 2 diabetes mellitus—mechanisms, management, and clinical considerations. Circulation. 2016;133(24):2459-2502. doi:10.1161/CIRCULATIONAHA.116.022194 27297342 PMC4910510

[2] Wu B, Bell K, Stanford A, . Understanding CKD among patients with T2DM: prevalence, temporal trends, and treatment patterns—NHANES 2007-2012. BMJ Open Diabetes Res Care. 2016;4(1):e000154. doi:10.1136/bmjdrc-2015-000154 27110365 PMC4838667

[3] United States Renal Data System. 2024 USRDS annual data report: epidemiology of kidney disease in the United States. Accessed December 30, 2024. https://www.usrds.org/annual-data-report/current-adr/

[4] Parker ED, Lin J, Mahoney T, . Economic costs of diabetes in the U.S. in 2022. Diabetes Care. 2024;47(1):26-43. doi:10.2337/dci23-0085 37909353

[5] Ndumele CE, Rangaswami J, Chow SL, ; American Heart Association. Cardiovascular-kidney-metabolic health: a presidential advisory from the American Heart Association. Circulation. 2023;148(20):1606-1635. doi:10.1161/CIR.0000000000001184 37807924

[6] Marso SP, Bain SC, Consoli A, ; SUSTAIN-6 Investigators. Semaglutide and cardiovascular outcomes in patients with type 2 diabetes. N Engl J Med. 2016;375(19):1834-1844. doi:10.1056/NEJMoa1607141 27633186

[7] Marso SP, Daniels GH, Brown-Frandsen K, ; LEADER Steering Committee; LEADER Trial Investigators. Liraglutide and cardiovascular outcomes in type 2 diabetes. N Engl J Med. 2016;375(4):311-322. doi:10.1056/NEJMoa1603827 27295427 PMC4985288

[8] Husain M, Birkenfeld AL, Donsmark M, ; PIONEER 6 Investigators. Oral semaglutide and cardiovascular outcomes in patients with type 2 diabetes. N Engl J Med. 2019;381(9):841-851. doi:10.1056/NEJMoa1901118 31185157

[9] Gerstein HC, Colhoun HM, Dagenais GR, ; REWIND Investigators. Dulaglutide and cardiovascular outcomes in type 2 diabetes (REWIND): a double-blind, randomised placebo-controlled trial. Lancet. 2019;394(10193):121-130. doi:10.1016/S0140-6736(19)31149-3 31189511

[10] Hegland TA, Fang Z, Bucher K. GLP-1 medication use for type 2 diabetes has soared. JAMA. 2024;332(12):952-953. doi:10.1001/jama.2024.18219 39212980

[11] Hamed K, Alosaimi MN, Ali BA, . Glucagon-like peptide-1 (GLP-1) receptor agonists: exploring their impact on diabetes, obesity, and cardiovascular health through a comprehensive literature review. Cureus. 2024;16(9):e68390. doi:10.7759/cureus.68390 39355484 PMC11444311

[12] Perkovic V, Tuttle KR, Rossing P, ; FLOW Trial Committees and Investigators. Effects of semaglutide on chronic kidney disease in patients with type 2 diabetes. N Engl J Med. 2024;391(2):109-121. doi:10.1056/NEJMoa2403347 38785209

[13] Alfayez OM, Almohammed OA, Alkhezi OS, Almutairi AR, Al Yami MS. Indirect comparison of glucagon like peptide-1 receptor agonists regarding cardiovascular safety and mortality in patients with type 2 diabetes mellitus: network meta-analysis. Cardiovasc Diabetol. 2020;19(1):96. doi:10.1186/s12933-020-01070-z 32571416 PMC7310317

[14] Dai JW, Lin Y, Li XW, . Comparative cardiovascular and renal outcomes of liraglutide versus dulaglutide in Asian type 2 diabetes patients. Sci Rep. 2024;14(1):27491. doi:10.1038/s41598-024-79255-9 39528690 PMC11555252

[15] US Department of Veterans Affairs. National Center for Veterans Analysis and Statistics. Accessed July 26, 2024. https://www.va.gov/vetdata/

[16] US Department of Veterans Affairs. Veterans Affairs Informatics and Computing Infrastructure (VINCI). Accessed July 26, 2024. https://www.research.va.gov/programs/vinci/default.cfm

[17] US Department of Veterans Affairs. VIReC research user guides. Accessed July 26, 2024. https://www.virec.research.va.gov/Resources/RUGs.asp

[18] Hernán MA, Robins JM. Using big data to emulate a target trial when a randomized trial is not available. Am J Epidemiol. 2016;183(8):758-764. doi:10.1093/aje/kwv254 26994063 PMC4832051

[19] Lund JL, Richardson DB, Stürmer T. The active comparator, new user study design in pharmacoepidemiology: historical foundations and contemporary application. Curr Epidemiol Rep. 2015;2(4):221-228. doi:10.1007/s40471-015-0053-5 26954351 PMC4778958

[20] Yoshida K, Solomon DH, Kim SC. Active-comparator design and new-user design in observational studies. Nat Rev Rheumatol. 2015;11(7):437-441. doi:10.1038/nrrheum.2015.30 25800216 PMC4486631

[21] Shen J, Sarwal A, Singh R, . Comparative effectiveness of insulin glargine, GLP-1RA and SGLT2i in veterans with type 2 diabetes. Diabetes Obes Metab. 2025;27(4):2120-2130. doi:10.1111/dom.16207 39887855 PMC11885103

[22] Liaw W, Adepoju OE, Luo J, . Factors Associated with Health Care Costs in Older Adults with Type 2 Diabetes: Insights for Value-Based Payment Models. Popul Health Manag. 2025;28(4):191-197. doi:10.1089/pop.2025.0054 40401431

[23] Lamprea-Montealegre JA, Madden E, Tummalapalli SL, . Association of Race and Ethnicity With Prescription of SGLT2 Inhibitors and GLP1 Receptor Agonists Among Patients With Type 2 Diabetes in the Veterans Health Administration System. JAMA. 2022;328(9):861-871. doi:10.1001/jama.2022.13885 36066519 PMC9449794

[24] Inker LA, Eneanya ND, Coresh J, ; Chronic Kidney Disease Epidemiology Collaboration. New creatinine- and cystatin C–based equations to estimate GFR without race. N Engl J Med. 2021;385(19):1737-1749. doi:10.1056/NEJMoa2102953 34554658 PMC8822996

[25] Zhang Z, Kim HJ, Lonjon G, Zhu Y; AME Big-Data Clinical Trial Collaborative Group. Balance diagnostics after propensity score matching. Ann Transl Med. 2019;7(1):16. doi:10.21037/atm.2018.12.10 30788363 PMC6351359

[26] Hernán MA, Hernández-Díaz S. Beyond the intention-to-treat in comparative effectiveness research. Clin Trials. 2012;9(1):48-55. doi:10.1177/1740774511420743 21948059 PMC3731071

[27] Hernán MA, Robins JM. Per-Protocol Analyses of Pragmatic Trials. N Engl J Med. 2017;377(14):1391-1398. doi:10.1056/NEJMsm1605385 28976864

[28] Diggle P. Analysis of Longitudinal Data. 2nd ed. Oxford University Press; 2013.

[29] Cole SR, Hernán MA, Margolick JB, Cohen MH, Robins JM. Marginal structural models for estimating the effect of highly active antiretroviral therapy initiation on CD4 cell count. Am J Epidemiol. 2005;162(5):471-478. doi:10.1093/aje/kwi216 16076835

[30] Drucker DJ. Prevention of cardiorenal complications in people with type 2 diabetes and obesity. Cell Metab. 2024;36(2):338-353. doi:10.1016/j.cmet.2023.12.018 38198966

[31] Drucker DJ. Mechanisms of action and therapeutic application of glucagon-like peptide-1. Cell Metab. 2018;27(4):740-756. doi:10.1016/j.cmet.2018.03.001 29617641

[32] Nauck MA, Meier JJ. Management of endocrine disease: are all GLP-1 agonists equal in the treatment of type 2 diabetes? Eur J Endocrinol. 2019;181(6):R211-R234. doi:10.1530/EJE-19-0566 31600725

[33] Mann JFE, Ørsted DD, Brown-Frandsen K, ; LEADER Steering Committee and Investigators. Liraglutide and renal outcomes in type 2 diabetes. N Engl J Med. 2017;377(9):839-848. doi:10.1056/NEJMoa1616011 28854085

[34] Knudsen LB, Lau J. The discovery and development of liraglutide and semaglutide. Front Endocrinol (Lausanne). 2019;10:155. doi:10.3389/fendo.2019.00155 31031702 PMC6474072

[35] Scheen AJ. Dulaglutide (LY-2189265) for the treatment of type 2 diabetes. Expert Rev Clin Pharmacol. 2016;9(3):385-399. doi:10.1586/17512433.2016.1141046 26761217

[36] Barrington P, Chien JY, Showalter HDH, . A 5-week study of the pharmacokinetics and pharmacodynamics of LY2189265, a novel, long-acting glucagon-like peptide-1 analogue, in patients with type 2 diabetes. Diabetes Obes Metab. 2011;13(5):426-433. doi:10.1111/j.1463-1326.2011.01364.x 21251178

[37] Frías JP, Davies MJ, Rosenstock J, ; SURPASS-2 Investigators. Tirzepatide versus semaglutide once weekly in patients with type 2 diabetes. N Engl J Med. 2021;385(6):503-515. doi:10.1056/NEJMoa2107519 34170647

[38] Yao H, Zhang A, Li D, . Comparative effectiveness of GLP-1 receptor agonists on glycaemic control, body weight, and lipid profile for type 2 diabetes: systematic review and network meta-analysis. BMJ. 2024;384:e076410. doi:10.1136/bmj-2023-076410 38286487 PMC10823535

[39] Aronne LJ, Horn DB, le Roux CW, ; SURMOUNT-5 Trial Investigators. Tirzepatide as compared with semaglutide for the treatment of obesity. N Engl J Med. 2025;393(1):26-36. doi:10.1056/NEJMoa2416394 40353578

[40] Luo J, Feldman R, Rothenberger SD, Hernandez I, Gellad WF. Coverage, formulary restrictions, and out-of-pocket costs for sodium-glucose cotransporter 2 inhibitors and glucagon-like peptide 1 receptor agonists in the Medicare Part D program. JAMA Netw Open. 2020;3(10):e2020969. doi:10.1001/jamanetworkopen.2020.20969 33057641 PMC7563069

[41] Whitley HP, Trujillo JM, Neumiller JJ. Special report: potential strategies for addressing GLP-1 and dual GLP-1/GIP receptor agonist shortages. Clin Diabetes. 2023;41(3):467-473. doi:10.2337/cd23-0023 37456085 PMC10338283

