# Supplementary material for: Liraglutide vs Semaglutide vs Dulaglutide in Veterans With Type 2 Diabetes
Source: JAMA Netw Open. 2025 Oct 13;8(10):e2537297. doi: 10.1001/jamanetworkopen.2025.37297 (PMC12519307; doi:10.1001/jamanetworkopen.2025.37297)
Supplement: Supplement 1. — eMethods. eTable 1. Definitions of baseline variables eTable 2. Definitions of study outcomes eTable 3. Missingness of baseline characteristics overall and by treatment group eTable 4. Median follow-up time in the overall cohort by outcome eTable 5. Incidence rates for the effectiveness outcomes among veterans initiating the study drugs eTable 6. Absolute risk, absolute risk differences, and risk ratios at 3 years between the treatment groups for each outcome of interest eTable 7. Sensitivity analysis removing index year from the propensity score model eTable 8. 12-Month treatment discontinuation and switch rates among study cohort with 1 complete year of follow-up data eTable 9. Reasons for censoring at each 3-month interval in the per-protocol analysis (protocol A, adhering for at least 45 days during each interval) eTable 10. Reasons for censoring at each 3-month interval in the per-protocol analysis (protocol B, adhering for at least 1 day during each interval) eTable 11. Weighted per-protocol event rates for the effectiveness and safety outcomes among veterans initiating the study drugs eTable 12. Intent-to-treat gastrointestinal adverse events among veterans initiating the study drugs eTable 13. Comparing the weighted intent-to-treat and per-protocol hazard ratios for the effectiveness outcomes among veterans initiating the study drugs eFigure 1. Flowchart of veteran selection eFigure 2. Histograms of the propensity score by treatment group eFigure 3. Balance of baseline characteristics before and after weighting eFigure 4. Weight change at 24 months among Veterans initiating liraglutide, semaglutide, and dulaglutide eFigure 5. Subgroup results eReferences. [file jamanetwopen-e2537297-s001.pdf]

Supplemental Online Content

Derington CG, Sarwal A, Wei G, et al. Liraglutide vs semaglutide vs dulaglutide in veterans with type 2 diabetes. *JAMA Netw Open*. 2025;8(10):e2537297. doi:10.1001/jamanetworkopen.2025.37297

eMethods.

eTable 1. Definitions of baseline variables

eTable 2. Definitions of study outcomes

eTable 3. Missingness of baseline characteristics overall and by treatment group

eTable 4. Median follow-up time in the overall cohort by outcome

eTable 5. Incidence rates for the effectiveness outcomes among veterans initiating the study drugs

eTable 6. Absolute risk, absolute risk differences, and risk ratios at 3 years between the treatment groups for each outcome of interest

eTable 7. Sensitivity analysis removing index year from the propensity score model

eTable 8. 12-Month treatment discontinuation and switch rates among study cohort with 1 complete year of follow-up data

eTable 9. Reasons for censoring at each 3-month interval in the per-protocol analysis (protocol A, adhering for at least 45 days during each interval)

eTable 10. Reasons for censoring at each 3-month interval in the per-protocol analysis (protocol B, adhering for at least 1 day during each interval)

© 2025 Derington CG et al. *JAMA Network Open*.

eTable 11. Weighted per-protocol event rates for the effectiveness and safety outcomes among veterans initiating the study drugs

eTable 12. Intent-to-treat gastrointestinal adverse events among veterans initiating the study drugs

eTable 13. Comparing the weighted intent-to-treat and per-protocol hazard ratios for the effectiveness outcomes among veterans initiating the study drugs

eFigure 1. Flowchart of veteran selection

eFigure 2. Histograms of the propensity score by treatment group

eFigure 3. Balance of baseline characteristics before and after weighting

eFigure 4. Weight change at 24 months among Veterans initiating liraglutide, semaglutide, and dulaglutide

eFigure 5. Subgroup results

eReferences.

This supplemental material has been provided by the authors to give readers additional information about their work.

© 2025 Derington CG et al. *JAMA Network Open*.

eMethods.

Data sources

The data for this study were queried using the VA Informatics and Computing Infrastructure from the VA's Corporate Data Warehouse, VA-linked United States Renal Data System datafiles, fee-service files representing non-VA care, Managerial Cost Accounting, and Pharmacy Benefits Management.[1,2] Data were also included from the United States Veterans Eligibility Trends and Statistics file, which contains patient-reported sociodemographic information such as income and education. Rural-Urban Commuting Area codes from the Planning Systems Support Group data indicated rurality of the patient's home address.

Steps to Test for Proportional Hazards Assumption Violation

First, we performed Schoenfeld's test for non-proportional hazards for all 3 pairwise treatment comparisons. For this test, a global p-value of <0.05 would indicate a violation of the proportionality assumption. For all treatment comparisons and outcomes in this study, the global p-value was >0.05, indicating no violation of the proportionality assumption. This test is sensitive to sample size of the study, hence further checks are needed.

However, even if the linear time by treatment interaction considered by the Schoenfeld test is not statistically significant, the p-value of this test does not indicate the magnitude of the interaction. So, as a second step, we quantified the significance of the interaction term between treatment and categorical survival time periods, defined as 0-2 and 2+ years. This check is only performed among the primary comparison of interest, which is liraglutide vs. semaglutide. This this test, a p-value comparing individual treatment periods to one another would indicate a violation of the proportionality assumption. Results are shown in the table below.

Outcome of interest	Hazard Ratio (95% CI) Years 0-2	Hazard Ratio (95% CI) Years 2+	P-value Years 2+ vs. Years 0-2
Kidney failure	0.89 (0.46, 1.72)	0.96 (0.54, 1.74)	0.86
Kidney failure + death	0.78 (0.61, 0.99)	0.95 (0.75, 1.22)	0.24
CKM Composite	0.96 (0.81, 1.13)	0.96 (0.76, 1.20)	0.99
CKM Composite + death	0.93 (0.80, 1.07)	0.91 (0.75, 1.10)	0.88
MACE	0.95 (0.80, 1.13)	0.95 (0.76, 1.20)	0.99
MACE + death	0.92 (0.80, 1.07)	0.91 (0.75, 1.10)	0.90
Death	0.74 (0.58, 0.96)	0.94 (0.73, 1.23)	0.21
Gastroparesis	0.60 (0.36, 1.01)	0.85 (0.35, 2.02)	0.59
Intestinal obstruction	0.83 (0.54, 1.27)	0.95 (0.52, 1.73)	0.73
Gallstones	0.82 (0.58, 1.14)	0.79 (0.51, 1.22)	0.90
Acute cholecystitis	0.62 (0.33, 1.16)	1.10 (0.45, 2.70)	0.43
Acute pancreatitis	0.91 (0.53, 1.57)	0.73 (0.37, 1.44)	0.63

Per-protocol methods

In addition to the ITT analysis, we conducted a per-protocol analysis to evaluate the effects of continuous use of each GLP-1RA, accounting for adherence over time.[3,4] We applied inverse probability of treatment weighting (as in the primary ITT analysis), along with additional weights

© 2025 Derington CG et al. *JAMA Network Open*.

for time-varying nonadherence and non-administrative censoring. Time-varying covariates, adherence, and censoring status were updated in 3-month intervals. We tested two protocol definitions, described below. A list of time-varying covariates is included below. Missing covariates were handled using last observation carried forward.[5]

Adherence probabilities were modeled via pooled logistic regression, conditional on prior adherence and covariates measured before interval k, with natural cubic splines for time. Models were fit separately by treatment group. Stabilized weights for nonadherence and censoring were constructed with numerators based on baseline covariates. Final weights were the product of treatment, adherence, and censoring weights, truncated at the 0.1st and 99.9th percentiles.

We estimated adjusted odds ratios using weighted generalized estimating equations logistic models, assuming independence working correlation structure clustering at the patient level.[6] Models included splines for time and GLP-1RA indicators and were fit separately for each pairwise comparison: liraglutide vs. semaglutide, liraglutide vs. dulaglutide, and dulaglutide vs. semaglutide.

Protocols evaluated

Nonadherence during interval *k* was defined as <*x* days of exposure to the index GLP-1RA, switching to another GLP-1RA, or missing drug data.

In protocol A, *x* was defined as 45 days (i.e., a patient was must have at least 45 days of medication on-hand, or <50% of the interval, to be considered adherent). We separately evaluated a protocol B setting *x to* 1 (i.e., a patient had to have at least 1 day of medication on-hand to be considered adherent). Contrasting the two protocols results in a pseudo-dose effect comparison, whereby protocol B indicates a lower dose, and protocol A indicates a higher dose.

Time-varying covariates

The following covariates were assessed during each 3-month interval.

- Mean systolic blood pressure during the interval.
- Body mass index during the interval.
- Mean hemoglobin A1c during the interval.
- Mean estimated glomerular filtration rate.
- Use of any insulin medication for ≥1 day during the interval.
- Use of any sodium-glucose co-transporter 2 inhibitor during the interval.
- Total sum of diabetes medications used during the interval.
- Total sum of non-diabetes medications (i.e., aspirin + statin + antihypertensives) used during the interval.
- Serious or transient side effects occurring during the interval assessed with diagnosis codes.
  - Serious: serious hypoglycemic episode, diabetic ketoacidosis episode, gastroparesis, paralytic ileus, intestinal obstruction, gallstones, acute cholecystitis, chronic and other cholecystitis, acute pancreatitis, chronic pancreatitis, other pancreas disorders, or malignant neoplasm of the pancreas.
  - Transient: nausea with or without vomiting, injection site reaction, non-aspecific adverse effect.
- Microvascular complications (i.e., diabetic neuropathy or retinopathy) occurring during the interval assessed with diagnosis codes.

© 2025 Derington CG et al. *JAMA Network Open*.

Weight loss analyses

We used linear mixed effect models to estimate the mean weight changes (lb) and the geometric mean percent change from baseline to follow-up at 6-month intervals through month 24 within and between the treatment groups. The applied an unstructured covariance model to account for serial correlation and used empirical standard errors for statistical inference. Inverse probability of treatment weighting was re-constructed and applied to balance the baseline variables across the treatment groups among patients with non-missing baseline body weight within the one-year pre-index period (N=20404, 94% of the primary analytic cohort). The follow-up weights were defined by the average of all weights measured in outpatient settings across each 6-month follow-up interval after the index date.

© 2025 Derington CG et al. *JAMA Network Open*.

eTable 1. Definitions of baseline variables.

Variable	Definition	Included in propensity score estimation
Index date and year	Date of the first initiation of liraglutide, semaglutide, or dulaglutide.	Yes in primary analysis, no in sensitivity analysis
Age	Age of the patient in years on the index date, defined using the date of birth on file based on the SPatient datafile.	Yes
Sex	Defined as male or female based on the SPatient datafile.	Yes
Race	Self-identified race based on USVETS datafiles, defined as White, Black/African American, Other or Mixed Races, or Unknown.	Yes
Ethnicity	Self-identified ethnicity based on USVETS datafiles, defined as Hispanic, Non-Hispanic, or Unknown.	Yes
Household income	Patient's household income level based on self-reported data in the USVETS datafiles. May be null if not available.	Yes
Education (individual)	Patient's education level based on self-reported data in the USVETS datafiles. May be null if not available.	Yes
Education (household)	Patient's household education level based on self-reported data in the USVETS datafiles. May be null if not available.	Yes
Rurality – rural versus urban	Based on Rural-Urban Commuting Area (RUCA) codes derived from the Planning Systems Support Group (PSSG) datafiles. RUCA codes identifying rural (including highly rural) include: 2.0, 2.1, 3.0, 4.0, 4.1, 5.0, 5.1, 6.0, 7.0, 7.1, 7.2, 8.0, 8.1, 8.2, 9.0, 10.0, 10.1, 10.2, 10.3 Missing RUCA codes also will identify a veteran as living in a rural or highly rural area, as they indicate that a patient was living on an island. RUCA codes identifying urban include: 1.0 and 1.1.	Yes
Systolic blood pressure	Mean of the three closest outpatient systolic blood pressure readings to the index date, within 365 days prior to index date. Expressed as mm Hg. Range: [70, 250].	Yes
Diastolic blood pressure	Mean of the three closest outpatient diastolic blood pressure readings to the index date, within 365 days prior to index date. Expressed as mm Hg. Range: [20, 130].	Yes
Systolic blood pressure count	Number of days in the 365 days prior to the index period with measured outpatient systolic blood pressure measurements.	Yes
Body mass index	Calculated as weight (kg) divided by height (m^2). Weight measurements were derived from outpatient measurements within 365 days prior to index date. Height measurements were derived from any value obtained prior to the index date or 90 days after the index date in the inpatient or outpatient setting. Missing if height or weight was missing, or if values were outside of range: [10, 75].	Yes
Hemoglobin A1c	Expressed as a percentage and derived from outpatient labs within 365 days prior to index date. Range: [0, 25].	Yes

© 2025 Derington CG et al. *JAMA Network Open*.

Estimated glomerular filtration rate	Calculated using the CKD EPI 2021 equation using outpatient serum creatinine labs within 365 days prior to index date. Expressed as mL/min/1.73m^2. Range: [0, 250].	Yes
Urine albumin-to-creatinine ratio	Expressed as mg/g and derived from outpatient labs within 365 days prior to index date. Range: [0, 50000].	Yes
Duration of type 2 diabetes (T2D) diagnosis	Calculated as the time difference between index date and the earliest date among the following three date,<br>1. First date of A1C > 6.4 between 01-01-1999 and index date.<br>2. Release date time for the first antidiabetic outpatient meds between 01-01-2005 and index date.<br>3. Diagnosis date of the first T2D ICD9/ICD10 codes between 01-01-1999 and index date. ICD9 codes included 150._0 and 250._2, and ICD10 code included E11.%.	Yes
Diabetic retinopathy	Presence of one of the following diagnosis codes from 01/01/2000 to the index date (dates inclusive) in outpatient or inpatient datafiles.<br>ICD-9: 362.0x<br>ICD-10: E08.3x, E09.3x, E11.3x, E13.3x	Yes
Diabetic neuropathy	Presence of one of the following diagnosis codes from 01/01/2000 to the index date (dates inclusive) in outpatient or inpatient datafiles.<br>ICD-9: 357.2<br>ICD-10: E08.40, E08.43, E09.40, E09.43, E11.40, E11.43, E13.40, E13.43	Yes
Serious hypoglycemic episodes	Presence of an episode of serious hypoglycemia, based on the presence of one of the following diagnosis codes from 01/01/2000 to the index date (dates inclusive) in outpatient or inpatient datafiles.<br>ICD-9: 251.x<br>ICD-10: E16.0x-16.2x	Yes
Coronary artery disease	Presence of one of the following diagnosis codes from 01/01/2000 to the index date (dates inclusive) in outpatient or inpatient datafiles.<br>ICD-9: 410.x,411.x, 412.x-414.x, V45.81, V45.82; or ICD-9 procedure code 36.x<br>ICD-10: I20-I22, I25.1, I25.2, I25.6-I25.9, T82.855A, T82.855D, T82.855S, Z95.1, Z95.5, Z98.61	Yes
Heart failure	Presence of one of the following diagnosis codes from 01/01/2000 to the index date (dates inclusive) in outpatient or inpatient datafiles.<br>ICD-9: 398.91, 402.x1, 404.x1, 404.x3, 428.x<br>ICD-10: I09.81, I11.0, I13.0, I13.2, I50.x, P29.0	Yes
Cardiomyopathy	Presence of one of the following diagnosis codes from 01/01/2000 to the index date (dates inclusive) in outpatient or inpatient datafiles.<br>ICD-9: 402.x0, 425.x, 429.83, 674.5x<br>ICD-10: A36.81, I11.x, I25.5, I42.x, I43, I51.81, O90.3	Yes
Cerebrovascular disease	Presence of one of the following diagnosis codes from 01/01/2000 to the index date (dates inclusive) in outpatient or inpatient datafiles.	Yes

© 2025 Derington CG et al. *JAMA Network Open*.

	ICD-9: 362.34, 430.x-438.x<br>ICD-10: G45.x, G46.x, H34.0, I60.x-I69.x	
Peripheral vascular disease	Presence of one of the following diagnosis codes from 01/01/2000 to the index date (dates inclusive) in outpatient or inpatient datafiles.<br>ICD-9: 093.0, 437.3, 440.x, 441.x, 443.x, 447.1, 557.1, 557.9, V43.4<br>ICD-10: I70.x, I71.x, I73.1, I73.8, I73.9, I77.1, I79.0, I79.2, K55.1, K55.8, K55.9, Z95.8, Z95.9	Yes
Hypertension	Presence of one of the following diagnosis codes from 01/01/2000 to the index date (dates inclusive) in outpatient or inpatient datafiles.<br>ICD-9: 401.x, 405.x<br>ICD-10: I10.x, I15.x, I16.x	Yes
Atrial fibrillation/flutter	Presence of one of the following diagnosis codes from 01/01/2000 to the index date (dates inclusive) in outpatient or inpatient datafiles.<br>ICD-9: 427.3x<br>ICD-10: I48.x	Yes
Chronic lung diseases/sleep apnea	Presence of one of the following diagnosis codes from 01/01/2000 to the index date (dates inclusive) in outpatient or inpatient datafiles.<br>ICD-9: 327.2x, 416.8, 416.9, 490.x-505.x, 506.4, 508.1, 508.8<br>ICD-10: G47.3x, I27.8x, I27.9, J40.x-J47.x, J60.x-J67.x, J68.4, J70.1, J70.3	Yes
Cancer	Presence of one of the following diagnosis codes from 01/01/2000 to the index date (dates inclusive) in outpatient or inpatient datafiles.<br>ICD-9: 140.x-172.x, 174.x-208.x, 238.6<br>ICD-10: C00.x- C43.x, C45.x- C80.x, C81.x-C85.x, C88.x-C96.x	Yes
HIV/AIDS	Presence of one of the following diagnosis codes from 01/01/2000 to the index date (dates inclusive) in outpatient or inpatient datafiles.<br>ICD-9: 042.x-044.x<br>ICD-10: B20.x-B22.x, B24.x	Yes
Traumatic brain injury	Presence of one of the following diagnosis codes from 01/01/2000 to the index date (dates inclusive) in outpatient or inpatient datafiles.<br>ICD-9: 850.x- 853.x<br>ICD-10: S06.0x-S06.3x, S06.5x-S06.6x, S06.Ax	Yes
Post-traumatic stress disorder	Presence of one of the following diagnosis codes from 01/01/2000 to the index date (dates inclusive) in outpatient or inpatient datafiles.<br>ICD-9: 309.81<br>ICD-10: F43.1x	Yes
Depression	Presence of one of the following diagnosis codes from 01/01/2000 to the index date (dates inclusive) in outpatient or inpatient datafiles.<br>ICD-9: 296.2x, 296.3x, 311<br>ICD-10: F32.x, F33.x	Yes

© 2025 Derington CG et al. *JAMA Network Open*.

Generalized anxiety disorder	Presence of one of the following diagnosis codes from 01/01/2000 to the index date (dates inclusive) in outpatient or inpatient datafiles.<br>ICD-9: 300.02<br>ICD-10: F41.1, F41.9	Yes
Tobacco use	Presence of one of the following diagnosis codes from 01/01/2000 to the index date (dates inclusive) in outpatient or inpatient datafiles.<br>ICD-9: 305.1x<br>ICD-10: F17.2x	Yes
Alcohol use	Presence of one of the following diagnosis codes from 01/01/2000 to the index date (dates inclusive) in outpatient or inpatient datafiles.<br>ICD-9: 305.0x<br>ICD-10: F10.x	Yes
Cannabis Use	Presence of one of the following diagnosis codes from 01/01/2000 to the index date (dates inclusive) in outpatient or inpatient datafiles.<br>ICD-9: 305.2x<br>ICD-10: F12.x	Yes
Other substance use	Presence of substance use other than tobacco, alcohol, or cannabis, based on one of the following diagnosis codes from 01/01/2000 to the index date (dates inclusive) in outpatient or inpatient datafiles.<br>ICD-9: 305.3x-305.9x<br>ICD-10: F11.x, F13.x, F14.x, F16.x	Yes
History of GI conditions	Presence of a history of GI conditions, including gastroparesis, paralytic ileus, intestinal obstruction, gallstones, acute cholescystitis, chronic and other cholecystitis, acute pancreatitis, chronic pancreatitis, other pancreas disorders, or malignant neoplasm of the pancreas. Based on one of the following diagnosis codes from 01/01/2000 to the index date (dates inclusive) in outpatient or inpatient datafiles.<br>ICD-9: 577.2, 577.8, 577.9, 157, 560.1, 577.0, 577.1, 537.3, 560.0, 560.2, 560.3x, 560.81, 560.89, 560.9, 564.7,564.81, 536.3, 574.x, 574.0x, 574.3x, 574.6x, 574.8x 575.0, 575.12, 574.1x, 574.4x, 574.7x, 574.8x, 575.1x<br>ICD-10: K86.2, K86.3, 86.8x, K86.9, C25, K56.0, K85.x, K86.0, K86.1, K31.5, K56.1, K56.2, K56.3, K56.4x,K56.5x, K56.6x, K56.7, K59.2, K59.3x, K31.84, K80.x, K81.0, K81.2, K80.0x, K80.12, K80.13, K80.42, K80.43, K80.46, K80.47,K80.62, K80.63, K80.66, K80.67, K81.1, K81.2, K81.9, K80.1x<br>K80.40, K80.41, K80.44, K80.45, K80.46, K80.47, K80.60, K80.61, K80.64, K80.65, K80.66, K80.67	Yes
Dipeptidyl Peptidase IV Inhibitors	Use of one of the following medications in the 180-day pre-index period:<br>Alogliptin, Linagliptin, Saxagliptin, Sitagliptin	Yes
Sulfonylureas	Use of one of the following medications in the 180-day pre-index period:<br>Acetohexamide, Chlorpropamide, Glimepiride, Glipizide, Glyburide, Tolazamide Tolbutamide	Yes

© 2025 Derington CG et al. *JAMA Network Open*.

Meglitinides	Use of one of the following medications in the 180-day pre-index period:<br>Nateglinide, Repaglinide	Yes
Thiazolidinediones	Use of one of the following medications in the 180-day pre-index period:<br>Pioglitazone, Rosiglitazone	Yes
Alpha glucosidase inhibitors	Use of one of the following medications in the 180-day pre-index period:<br>Acarbose, Miglitol	Yes
Insulin	Use of one of the following medications in the 180-day pre-index period:<br>Insulin lispro, insulin aspart, insulin glulisine, insulin regular, inhaled human insulin, Neutral Protamine Hagedorn insulin, insulin zinc formulations, insulin glargine, insulin detemir, or insulin degludec	Yes
Sodium glucose cotransporter 2 inhibitors	Use of one of the following medications in the 180-day pre-index period:<br>Bexagliflozin, Dapagliflozin, Canagliflozin, Empagliflozin, Sotagliflozin, Ertugliflozin	Yes
Other T2D classes	Use of one of the following medications in the 180-day pre-index period. Encompases amylinomimetics, dopamine 2 agonists, bile acid sequestrants.<br>Pramlintide, Bromocriptine, Colesevalam	Yes
Diuretic use	Use of one of the following medications in the 180-day pre-index period:<br>Acetazolamide, Amiloride, Bendroflumethiazide, Benzthiazide, Bumetanide, Chlorothiazide, Chlorthalidone, Cyclothiazide, Eplerenone, Ethacrynic Acid, Finerenone, Furosemide, Hydrochlorothiazide, Hydroflumethiazide, Indapamide, Methazolamide, Methyclothiazide, Metolazone, Polythiazide, Quinethazone, Spironolactone, Torsemide, Triamterene, Trichlormethiazide	Yes
Angiotensin-converting enzyme inhibitors/ Angiotensin-II receptor blockers	Use of one of the following medications in the 180-day pre-index period:<br>Benazepril, Captopril, Enalapril, Fosinopril, Lisinopril, Moexipril, Perindopril, Quinapril, Ramipril, Trandolapril, Azilsartan, Candesartan, Eprosartan, Irbesartan, Losartan, Olmesartan, Telmisartan, Valsartan	Yes
Alpha-blockers	Use of one of the following medications in the 180-day pre-index period:<br>Alfuzosin, Doxazosin, Prazosin, Silodosin, Tamsulosin, Terazosin	Yes
Beta-blockers	Use of one of the following ORAL medications in the 180-day pre-index period:<br>Acebutolol, Atenolol, Betaxolol, Bisoprolol, Carvedilol, Labetalol, Metoprolol, Nadolol, Nebivolol, Penbutolol, Pindolol, Propranolol, Timolol	Yes
Calcium Channel Blockers	Use of one of the following ORAL medications in the 180-day pre-index period:<br>Amlodipine, Diltiazem, Felodipine, Isradipine, Nicardipine, Nifedipine, Nimodipine, Nisoldipine, Verapamil	Yes
Alpha-2 agonists or central agents	Use of one of the following medications in the 180-day pre-index period:<br>Clonidine (oral or patch), Guanabenz, Guanfacine, Methyldopa, Reserpine, Deserpidine, Guanethidine, Guanadrel	Yes
Vasodilators	Use of one of the following medications in the 180-day pre-index period:<br>Hydralazine, Minoxidil	Yes
Mineralocorticoid Receptor Antagonists	Use of one of the following medications in the 180-day pre-index period:<br>Finerenone, Spironolactone, Eplerenone	Yes

© 2025 Derington CG et al. *JAMA Network Open*.

Statins	Use of one of the following medications:<br>Atorvastatin, Cerivastatin, Rosuvastatin, Simvastatin, Fluvastatin, Lovastatin, Pitavastatin, Pravastatin	Yes

© 2025 Derington CG et al. *JAMA Network Open*.

eTable 2. Definitions of study outcomes.

Outcome	Definition
Kidney failure	Dichotomous variable indicating the post-index occurrence of one of the composite events:<br>1) CKD Stage V, defined as: Sustained eGFR <15 mL/min/1.73m^2 calculated using the CKD EPI 2021 equation across 2 consecutive laboratory measurements (outpatient or inpatient) spaced at least 60 days apart. If second lab was not available due to loss to follow up, death, or end stage kidney disease, then the patient meets criteria for this outcome. If multiple values exist between the first and >60-day value, then all must be <15 mL/min/1.73m^2.<br>2) End-stage kidney disease (initiation of renal replacement therapy or pre-emptive kidney transplant) defined through the USRDS ESKD variable, VA diagnosis codes (Z94.0, Z48.22, T86.1x, N18.6), CPT codes 50360 or 50365, or a combination of diagnosis codes and CPT code 50360 or 50365.
All-cause death	Dichotomous variable indicating post-index death occurrence based on death data available in the Vital Status datafiles.
CVD events (aka “MACE”)	Dichotomous variable indicating the post-index occurrence of one of the composite event of myocardial infarction, heart failure, and stroke/TIA, as defined below.
Myocardial infarction	Dichotomous variable indicating post-index myocardial infarction occurrence based on presence of one of the following ICD-10 codes in inpatient, outpatient and fee basis datafiles: I21.x, I22.x, I25.6
Heart failure	Dichotomous variable indicating post-index heart failure occurrence based on presence of one of the following ICD-10 codes in inpatient, outpatient and fee basis datafiles: I11.0, I13.0, I13.2, I50.x
Stroke or transient ischemic attack (TIA)	Dichotomous variable indicating post-index stroke or TIA occurrence based on presence of one of the following ICD-10 codes in inpatient, outpatient and fee basis datafiles: I600-I616, I618, I619, I630-I636, I638, I639, I64, G450-459
CKM composite	Dichotomous variable indicating the post-index occurrence of one of the composite events:<br>1) CKD Stage V, defined as: Sustained eGFR <15 mL/min/1.73m^2 calculated using the CKD EPI 2021 equation across 2 consecutive laboratory measurements (outpatient or inpatient) spaced at least 60 days apart. If second lab was not available due to loss to follow up, death, or end stage kidney disease, then the patient meets criteria for this outcome. If multiple values exist between the first and >60-day value, then all must be <15 mL/min/1.73m^2.<br>2) End-stage kidney disease (initiation of renal replacement therapy or pre-emptive kidney transplant) defined through the USRDS ESKD variable, VA diagnosis codes (Z94.0, Z48.22, T86.1x, N18.6), CPT codes 50360 or 50365, or a combination of diagnosis codes and CPT code 50360 or 50365.<br>3) CVD events as defined in the CVD event (MACE) composite (myocardial infarction, heart failure, and stroke/TIA).

© 2025 Derington CG et al. *JAMA Network Open*.

Gastroparesis	Dichotomous variable indicating post-index gastroparesis occurrence based on presence of one of the following ICD-10 codes in inpatient, outpatient and fee basis datafiles: K31.84
Intestinal obstructions	Dichotomous variable indicating post-index intestinal obstruction occurrence based presence of one of the following ICD-10 codes in inpatient, outpatient and fee basis datafiles: K56.3-gallstone ileus (intestinal obstruction)<br>K56.4X-other impaction of intestine, fecal impaction<br>K56.6X-unspecified and other intestinal obstruction<br>K59.3x-megacolon<br>K31.5-obstruction of duodenum<br>K56.1 - intussusception<br>K56.2 - volvulus<br>K56.5x - intestinal adhesions with obstruction<br>K56.7 - ileus<br>K59.2 - neurogenic bowel<br>K56.0-paralytic ileus
Gallstones	Dichotomous variable indicating post-index gallstones occurrence based on presence of one of the following ICD-10 codes in inpatient, outpatient and fee basis datafiles: K80.x
Acute Cholecystitis	Dichotomous variable indicating post-index acute cholescystitis occurrence based presence of one of the following ICD-10 codes in inpatient, outpatient and fee basis datafiles: K81.0, K81.2, K80.0x, K80.12, K80.13, K80.42, K80.43, K80.46, K80.47, K80.62, K80.63, K80.66, K80.67
Acute Pancreatitis	Dichotomous variable indicating post-index acute pancreatitis occurrence based presence of one of the following ICD-10 codes in inpatient, outpatient and fee basis datafiles: K85.x

© 2025 Derington CG et al. *JAMA Network Open*.

eTable 3. Missingness of baseline characteristics overall and by treatment group.

Note: characteristics present in Table 1 but not listed here have 0% missingness.

**Characteristic**	**Overall**<br>**N = 21,790**	**Dulaglutide**<br>**N = 5,527**	**Liraglutide**<br>**N = 5,425**	**Semaglutide**<br>**N = 10,838**
Race	19 (0.1%)	2 (0.0%)	7 (0.1%)	10 (0.1%)
Ethnicity	12 (0.1%)	1 (0.0%)	3 (0.1%)	8 (0.1%)
Individual Education	4,830 (22.2%)	1,191 (21.5%)	1,177 (21.7%)	2,462 (22.7%)
Household Education	375 (1.7%)	90 (1.6%)	89 (1.6%)	196 (1.8%)
Household income	375 (1.7%)	90 (1.6%)	89 (1.6%)	196 (1.8%)
Duration of type 2 diabetes disease	113 (0.5%)	8 (0.1%)	76 (1.4%)	29 (0.3%)
Systolic blood pressure	1,089 (5.0%)	172 (3.1%)	113 (2.1%)	804 (7.4%)
Diastolic blood pressure	1,091 (5.0%)	172 (3.1%)	114 (2.1%)	805 (7.4%)
Body Mass Index	1,545 (7.0%)	252 (4.6%)	164 (3.0%)	1,129 (10.4%)
Hemoglobin A1C	825 (3.8%)	221 (4.0%)	148 (2.7%)	456 (4.2%)
Estimated glomerular filtration rate	925 (4.2%)	266 (4.8%)	226 (4.2%)	433 (4.0%)
Urine albumin-to-creatinine ratio	9,409 (43.2%)	2,699 (48.8%)	2,387 (44.0%)	4,323 (39.9%)
Total cholesterol	2,215 (10.2%)	548 (9.9%)	490 (9.0%)	1,177 (10.9%)
High density lipoprotein cholesterol	1,964 (9.0%)	516 (9.3%)	415 (7.6%)	1,033 (9.5%)
Low density lipoprotein cholesterol	1,972 (9.1%)	523 (9.5%)	418 (7.7%)	1,031 (9.5%)
Triglycerides	2,030 (9.3%)	521 (9.4%)	439 (8.1%)	1,070 (9.9%)

Data are n and column proportions in parentheses, expressed as percentages.

© 2025 Derington CG et al. *JAMA Network Open*.

eTable 4. Median follow-up time in the overall cohort by outcome.

Outcome of interest	Median follow up time in overall cohort (in years)
Kidney Failure	2.99
Kidney Failure/Death	2.99
Death	3.01
MACE	2.75
CKM	2.74
MACE/Death	2.75
Gastroparesis	2.98
Intestinal Obstruction	2.97
Gallstones	2.95
Acute Cholecystitis	2.99
Acute Pancreatitis	2.98

CKM: cardio-kidney metabolic; MACE: major adverse cardiovascular events

© 2025 Derington CG et al. *JAMA Network Open*.

eTable 5: Incidence rates for the effectiveness outcomes among veterans initiating the study drugs.

	Unweighted			Inverse Probability Weighted		
**Outcome**	**Liraglutide N = 5,425**	**Semaglutide N = 10,838**	**Dulaglutide N = 5,527**	**Liraglutide**	**Semaglutide**	**Dulaglutide**
Kidney failure	0.38 (78/20427)	0.32 (82/25253)	0.33 (64/19236)	0.37	0.39	0.31
Kidney failure or death	2.45 (501/20427)	2.56 (647/25249)	2.92 (562/19236)	2.41	2.76	3.29
CKM composite	4.18 (793/18990)	4.14 (997/24105)	3.71 (675/18178)	4.01	4.18	4.41
CKM composite or death	5.61 (1065/18990)	5.76 (1388/24105)	5.64 (1025/18178)	5.48	5.91	6.56
MACE	4.01 (763/19026)	3.98 (962/24151)	3.53 (644/18220)	3.81	4.00	4.25
MACE or death	5.49 (1044/19026)	5.62 (1358/24151)	5.49 (1000/18220)	5.31	5.73	6.43
All-cause death	2.20 (451/20507)	2.35 (594/25322)	2.67 (516/19309)	2.13	2.50	3.05

Numbers in the “unweighted” columns are expressed as events per 100 person years (N events/years of follow-up). Numbers in the “weighted” columns are expressed as events per 100 person years.

CKM composite encompass a composite of CKD stage V, end-stage kidney disease, or MACE events. MACE is a composite of hospitalization for myocardial infarction, heart failure, and stroke/TIA.

© 2025 Derington CG et al. *JAMA Network Open*.

eTable 6. Absolute risk, absolute risk differences, and risk ratios at 3 years between the treatment groups for each outcome of interest.

Outcome	Inverse Probability Weighted								
	3-year absolute risks (95% CI)			3-year absolute risk differences (95% CI)			3-year risk ratio (95% CI)		
	Liraglutide	Semaglutide	Dulaglutide	Liraglutide vs. semaglutide	Liraglutide vs. dulaglutide	Dulaglutide vs. semaglutide	Liraglutide vs. semaglutide	Liraglutide vs. dulaglutide	Dulaglutide vs. semaglutide
Kidney failure	0.01 (0.01, 0.01)	0.01 (0.01, 0.02)	0.01 (0.01, 0.01)	0.00 (-0.01, 0.00)	0.00 (0.00, 0.01)	0.00 (-0.01, 0.00)	0.94 (0.57, 1.54)	1.17 (0.75, 1.83)	0.80 (0.50, 1.28)
Kidney failure or death	0.07 (0.06, 0.07)	0.08 (0.07, 0.09)	0.09 (0.08, 0.10)	-0.01 (-0.03, 0.00)	-0.03 (-0.04, -0.01)	0.01 (0.00, 0.03)	0.83 (0.69, 0.99)	0.71 (0.59, 0.86)	1.15 (0.96, 1.39)
CKM composite	0.11 (0.10, 0.12)	0.11 (0.10, 0.12)	0.12 (0.11, 0.14)	0.00 (-0.02, 0.01)	-0.01 (-0.03, 0.01)	0.01 (-0.01, 0.03)	1.00 (0.87, 1.14)	0.91 (0.78, 1.05)	1.10 (0.95, 1.28)
CKM composite or death	0.15 (0.14, 0.16)	0.16 (0.14, 0.17)	0.18 (0.16, 0.19)	-0.01 (-0.03, 0.01)	-0.03 (-0.05, -0.01)	0.02 (0.00, 0.04)	0.94 (0.84, 1.05)	0.83 (0.74, 0.93)	1.13 (1.01, 1.28)
MACE	0.11 (0.10, 0.12)	0.11 (0.10, 0.12)	0.12 (0.11, 0.13)	0.00 (-0.01, 0.02)	-0.01 (-0.03, 0.00)	0.01 (0.00, 0.03)	1.01 (0.88, 1.16)	0.89 (0.76, 1.03)	1.13 (0.97, 1.32)
MACE or death	0.14 (0.13, 0.16)	0.15 (0.14, 0.16)	0.18 (0.16, 0.19)	-0.01 (-0.03, 0.01)	-0.03 (-0.05, -0.01)	0.02 (0.00, 0.04)	0.94 (0.84, 1.06)	0.82 (0.72, 0.93)	1.15 (1.02, 1.31)
All-cause death	0.06 (0.05, 0.07)	0.07 (0.06, 0.08)	0.09 (0.07, 0.10)	-0.01 (-0.03, 0.00)	-0.03 (-0.04,-0.01)	0.01 (0.00, 0.03)	0.80 (0.66, 0.97)	0.67 (0.55, 0.83)	1.19 (0.97, 1.47)

CI: confidence interval; CKM: cardio-kidney metabolic; MACE: major adverse cardiovascular events

© 2025 Derington CG et al. *JAMA Network Open*.

eTable 7. Sensitivity analysis removing index year from the propensity score model.

Outcome	Liraglutide vs. Semaglutide Weighted HR (95% CI)		Liraglutide vs. Dulaglutide Weighted HR (95% CI)		Dulaglutide vs. Semaglutide Weighted HR (95% CI)	
	Primary analysis (index year included)	Sensitivity analysis (no index year)	Primary analysis (index year included)	Sensitivity analysis (no index year)	Primary analysis (index year included)	Sensitivity analysis (no index year)
Kidney Failure	0.93 (0.60, 1.44)	0.87 (0.61, 1.23)	1.19 (0.80, 1.77)	1.18 (0.83, 1.68)	0.80 (0.52, 1.23)	0.80 (0.56, 1.13)
Kidney Failure + death	0.85 (0.72, 1.01)	0.81 (0.71, 0.92)	0.73 (0.61, 0.87)	0.81 (0.71, 0.92)	1.18 (0.98, 1.41)	1.03 (0.91, 1.17)
CKM Composite	0.96 (0.84, 1.10)	0.92 (0.83, 1.02)	0.91 (0.78, 1.06)	1.03 (0.92, 1.15)	1.07 (0.91, 1.24)	0.94 (0.84, 1.04)
CKM Composite + death	0.92 (0.82, 1.03)	0.88 (0.81, 0.97)	0.83 (0.73, 0.95)	0.93 (0.85, 1.02)	1.11 (0.98, 1.27)	0.98 (0.90, 1.07)
MACE	0.95 (0.83, 1.09)	0.91 (0.82, 1.01)	0.90 (0.77, 1.05)	1.03 (0.92, 1.15)	1.07 (0.92, 1.26)	0.93 (0.84, 1.04)
MACE + death	0.92 (0.82, 1.03)	0.88 (0.81, 0.97)	0.82 (0.72, 0.94)	0.93 (0.84, 1.02)	1.12 (0.99, 1.28)	0.98 (0.90, 1.08)
Death	0.83 (0.69, 0.99)	0.79 (0.69, 0.91)	0.69 (0.58, 0.83)	0.77 (0.68, 0.89)	1.20 (0.99, 1.46)	1.04 (0.91, 1.18)
Gastroparesis	0.68 (0.44, 1.07)	0.73 (0.51, 1.03)	0.95 (0.64, 1.43)	0.83 (0.58, 1.20)	0.68 (0.45, 1.03)	0.83 (0.59, 1.15)
Intestinal obstruction	0.87 (0.62, 1.22)	0.81 (0.61, 1.06)	0.89 (0.60, 1.33)	1.00 (0.75, 1.33)	0.99 (0.67, 1.45)	0.81 (0.62, 1.05)
Gallstones	0.81 (0.62, 1.05)	0.86 (0.69, 1.06)	1.11 (0.83, 1.49)	1.15 (0.91, 1.45)	0.72 (0.54, 0.95)	0.76 (0.61, 0.95)
Acute cholecystitis	0.76 (0.47, 1.24)	0.87 (0.59, 1.26)	1.11 (0.69, 1.81)	1.13 (0.73, 1.75)	0.62 (0.39, 0.99)	0.69 (0.46, 1.03)
Acute pancreatitis	0.85 (0.55, 1.31)	0.91 (0.63, 1.29)	0.83 (0.48, 1.41)	0.92 (0.63, 1.33)	1.02 (0.59, 1.76)	0.97 (0.67, 1.39)

CI: confidence interval; CKM: cardio-kidney metabolic; CVD: cardiovascular disease; GI: gastrointestinal; HR: hazard ratio; MACE: major adverse cardiovascular event

© 2025 Derington CG et al. *JAMA Network Open*.

eTable 8. 12-month treatment discontinuation and switch rates among study cohort with 1 complete year of follow-up data.

Outcome	Overall N =21,235	Initial treatment group Liraglutide N = 5,326	Semaglutide N = 10,527	Dulaglutide N = 5,382
Taking index GLP-1RA at 12 months*	14,193 (66.8%)	3,242 (60.9%)	7,071 (67.2%)	3,880 (72.1%)
Switched to another GLP-1RA at any point in the first 12 months**	1,038 (4.9%)	449 (8.4%)	136 (1.3%)	453 (8.4%)
Number of days exposed to index GLP-1RA within the first 12 months of follow-up, mean (SD)[#]	262 (103)	235 (110)	273 (96)	267 (103)
Medication dose in follow-up, median [IQR]	--	1.5 mg daily (1.2, 1.8)	0.7 mg weekly (0.5, 0.9)	1.3 mg weekly (0.7, 1.5)

Numbers in the table are n (column percent).

* Patient meets criteria if, based on fill dates and days' supply, they have at least 50% of their 12th month of follow-up on-hand.

**Patient meets criteria if, based on dispense dates, they were dispensed another GLP-1RA at any point between day 0 and 365 of follow-up.

[#] Based on days' supply of all fills in the first year, rounded up to the nearest whole integer.

© 2025 Derington CG et al. *JAMA Network Open*.

eTable 9. Reasons for censoring at each 3-month interval in the per-protocol analysis (protocol A, adhering for at least 45 days during each interval).

			Censored during period			
**Index Treatment Group**	**Period (i.e., 3-month interval)**	**Number (%) at risk at start of period**	**Died**	**Lost to follow up**	**Violated protocol**	**Administratively censored during period**
Liraglutide	1	5425 (100)	12	6	725	0
Liraglutide	2	4682 (86.3)	14	2	1005	0
Liraglutide	3	3661 (67.5)	12	5	684	0
Liraglutide	4	2959 (54.5)	8	3	503	0
Liraglutide	5	2444 (45.1)	5	0	395	0
Liraglutide	6	2043 (37.7)	8	17	303	2
Liraglutide	7	1712 (31.6)	8	16	270	0
Liraglutide	8	1418 (26.1)	5	20	212	3
Liraglutide	9	1177 (21.7)	1	15	199	3
Liraglutide	10	959 (17.7)	2	9	176	2
Liraglutide	11	769 (14.2)	5	12	147	1
Liraglutide	12	604 (11.1)	2	6	133	0
Liraglutide	13	462 (8.5)	2	9	102	3
Liraglutide	14	345 (6.4)	1	5	68	3
Liraglutide	15	266 (4.9)	4	9	56	2
Liraglutide	16	195 (3.6)	0	8	35	6
Liraglutide	17	146 (2.7)	1	19	18	3
Liraglutide	18	105 (1.9)	1	13	12	3
Liraglutide	19	76 (1.4)	0	18	4	7
Liraglutide	20	47 (0.9)	0	23	3	4
Liraglutide	21	17 (0.3)	0	10	0	4

© 2025 Derington CG et al. *JAMA Network Open*.

Liraglutide	22	3 (0.1)	0	1	0	2
Liraglutide	23	0 (0)	0	0	0	0
Liraglutide	24	0 (0)	0	0	0	0
Semaglutide	1	10838 (100)	42	19	408	0
Semaglutide	2	10365 (95.6)	47	21	1848	0
Semaglutide	3	8441 (77.9)	34	20	865	0
Semaglutide	4	7521 (69.4)	22	17	576	0
Semaglutide	5	6904 (63.7)	29	53	606	0
Semaglutide	6	6215 (57.3)	16	645	425	134
Semaglutide	7	4993 (46.1)	19	598	339	113
Semaglutide	8	3921 (36.2)	14	499	272	125
Semaglutide	9	3009 (27.8)	6	431	176	101
Semaglutide	10	2291 (21.1)	5	310	136	76
Semaglutide	11	1762 (16.3)	7	159	101	33
Semaglutide	12	1462 (13.5)	8	115	95	25
Semaglutide	13	1218 (11.2)	12	156	78	24
Semaglutide	14	947 (8.7)	1	154	60	25
Semaglutide	15	706 (6.5)	1	156	51	29
Semaglutide	16	469 (4.3)	2	109	42	27
Semaglutide	17	289 (2.7)	0	111	19	23
Semaglutide	18	136 (1.3)	0	67	9	9
Semaglutide	19	51 (0.5)	0	42	2	4
Semaglutide	20	3 (0)	0	3	0	0
Semaglutide	21	0 (0)	0	0	0	0
Semaglutide	22	0 (0)	0	0	0	0
Semaglutide	23	0 (0)	0	0	0	0
Semaglutide	24	0 (0)	0	0	0	0
Dulaglutide	1	5527 (100)	33	5	443	0
Dulaglutide	2	5044 (91.3)	20	6	696	0
Dulaglutide	3	4322 (78.2)	22	7	509	0

© 2025 Derington CG et al. *JAMA Network Open*.

Dulaglutide	4	3783 (68.4)	12	4	420	0
Dulaglutide	5	3347 (60.6)	17	2	479	0
Dulaglutide	6	2848 (51.5)	12	7	384	4
Dulaglutide	7	2439 (44.1)	6	14	365	0
Dulaglutide	8	2053 (37.1)	6	15	294	4
Dulaglutide	9	1733 (31.4)	11	15	272	2
Dulaglutide	10	1431 (25.9)	3	32	281	5
Dulaglutide	11	1109 (20.1)	5	38	251	10
Dulaglutide	12	805 (14.6)	4	33	216	7
Dulaglutide	13	545 (9.9)	3	51	137	7
Dulaglutide	14	346 (6.3)	1	22	66	3
Dulaglutide	15	254 (4.6)	1	22	46	2
Dulaglutide	16	183 (3.3)	2	24	22	2
Dulaglutide	17	133 (2.4)	0	21	16	3
Dulaglutide	18	93 (1.7)	0	20	5	4
Dulaglutide	19	64 (1.2)	1	14	4	8
Dulaglutide	20	37 (0.7)	0	13	1	2
Dulaglutide	21	21 (0.4)	0	10	0	7
Dulaglutide	22	4 (0.1)	0	4	0	0
Dulaglutide	23	0 (0)	0	0	0	0
Dulaglutide	24	0 (0)	0	0	0	0

© 2025 Derington CG et al. *JAMA Network Open*.

eTable 10. Reasons for censoring at each 3-month interval in the per-protocol analysis (protocol B, adhering for at least 1 day during each interval).

			Censored during period			
**Index Treatment Group**	**Period (i.e., 3-month interval)**	**Number (%) at risk at start of period**	**Died**	**Lost to follow up**	**Violated protocol**	**Administratively censored during period**
Liraglutide	1	5425 (100)	12	6	65	0
Liraglutide	2	5342 (98.5)	19	4	1001	0
Liraglutide	3	4318 (79.6)	13	6	552	0
Liraglutide	4	3746 (69.1)	14	5	480	0
Liraglutide	5	3245 (59.8)	9	1	432	0
Liraglutide	6	2802 (51.6)	10	22	365	5
Liraglutide	7	2399 (44.2)	9	26	301	3
Liraglutide	8	2059 (38)	6	39	295	7
Liraglutide	9	1711 (31.5)	2	26	236	4
Liraglutide	10	1443 (26.6)	4	16	219	5
Liraglutide	11	1197 (22.1)	8	17	213	4
Liraglutide	12	955 (17.6)	4	13	184	0
Liraglutide	13	753 (13.9)	3	22	139	5
Liraglutide	14	583 (10.7)	2	13	103	4
Liraglutide	15	458 (8.4)	4	24	91	6
Liraglutide	16	333 (6.1)	1	19	50	6
Liraglutide	17	257 (4.7)	1	27	36	6
Liraglutide	18	187 (3.4)	2	21	18	8
Liraglutide	19	138 (2.5)	1	34	9	10
Liraglutide	20	84 (1.5)	0	37	1	12
Liraglutide	21	34 (0.6)	0	20	0	9

© 2025 Derington CG et al. *JAMA Network Open*.

			Censored during period			
Index Treatment Group	Period (i.e., 3-month interval)	Number (%) at risk at start of period	Died	Lost to follow up	Violated protocol	Administratively censored during period
Liraglutide	22	5 (0.1)	0	2	0	3
Liraglutide	23	0 (0)	0	0	0	0
Liraglutide	24	0 (0)	0	0	0	0
Semaglutide	1	10838 (100)	42	19	43	0
Semaglutide	2	10730 (99)	49	24	1095	0
Semaglutide	3	9554 (88.2)	46	26	668	0
Semaglutide	4	8813 (81.3)	28	21	435	0
Semaglutide	5	8325 (76.8)	35	74	355	0
Semaglutide	6	7860 (72.5)	28	868	306	188
Semaglutide	7	6466 (59.7)	25	859	222	155
Semaglutide	8	5202 (48)	20	766	186	177
Semaglutide	9	4050 (37.4)	8	630	128	130
Semaglutide	10	3150 (29.1)	7	450	86	107
Semaglutide	11	2498 (23)	12	225	69	54
Semaglutide	12	2137 (19.7)	13	189	65	36
Semaglutide	13	1832 (16.9)	16	273	48	43
Semaglutide	14	1450 (13.4)	4	248	32	50
Semaglutide	15	1115 (10.3)	2	257	35	49
Semaglutide	16	771 (7.1)	3	198	28	44
Semaglutide	17	498 (4.6)	1	206	9	43
Semaglutide	18	239 (2.2)	0	120	5	21
Semaglutide	19	93 (0.9)	0	75	0	12
Semaglutide	20	6 (0.1)	0	6	0	0

© 2025 Derington CG et al. *JAMA Network Open*.

			Censored during period			
Index Treatment Group	Period (i.e., 3-month interval)	Number (%) at risk at start of period	Died	Lost to follow up	Violated protocol	Administratively censored during period
Semaglutide	21	0 (0)	0	0	0	0
Semaglutide	22	0 (0)	0	0	0	0
Semaglutide	23	0 (0)	0	0	0	0
Semaglutide	24	0 (0)	0	0	0	0
Dulaglutide	1	5527 (100)	33	5	31	0
Dulaglutide	2	5456 (98.7)	21	9	631	0
Dulaglutide	3	4795 (86.8)	25	10	478	0
Dulaglutide	4	4278 (77.4)	13	6	413	0
Dulaglutide	5	3846 (69.6)	21	3	434	0
Dulaglutide	6	3386 (61.3)	13	15	399	5
Dulaglutide	7	2952(53.4)	10	19	383	0
Dulaglutide	8	2539(45.9)	11	22	332	4
Dulaglutide	9	2169(39.2)	13	24	299	5
Dulaglutide	10	1826(33)	4	45	318	8
Dulaglutide	11	1450(26.2)	6	53	292	12
Dulaglutide	12	1087(19.7)	5	42	260	8
Dulaglutide	13	771(13.9)	7	66	191	12
Dulaglutide	14	493(8.9)	2	38	88	3
Dulaglutide	15	362(6.5)	2	36	54	3
Dulaglutide	16	267(4.8)	2	33	24	4
Dulaglutide	17	204(3.7)	0	36	17	4
Dulaglutide	18	147(2.7)	0	33	8	6
Dulaglutide	19	100(1.8)	2	25	6	9

© 2025 Derington CG et al. *JAMA Network Open*.

			Censored during period			
Index Treatment Group	Period (i.e., 3-month interval)	Number (%) at risk at start of period	Died	Lost to follow up	Violated protocol	Administratively censored during period
Dulaglutide	20	58(1)	0	27	0	2
Dulaglutide	21	29(0.5)	0	15	1	9
Dulaglutide	22	4(0.1)	0	4	0	0
Dulaglutide	23	0(0)	0	0	0	0
Dulaglutide	24	0(0)	0	0	0	0

© 2025 Derington CG et al. *JAMA Network Open*.

eTable 11. Weighted per-protocol event rates for the effectiveness and safety outcomes among veterans initiating the study drugs.

	Liraglutide			Semaglutide			Dulaglutide		
**Outcome**	**ITT**	**PP – ≥45 day**	**PP – ≥1 day**	**ITT**	**PP – ≥45 day**	**PP – ≥1 day**	**ITT**	**PP – ≥45 day**	**PP – ≥1 day**
Effectiveness outcomes									
Kidney failure	0.37	0.21	0.27	0.39	0.37	0.34	0.31	0.21	0.25
Kidney failure or death	2.41	1.60	1.76	2.76	1.88	2.03	3.29	2.98	2.81
CKM composite	4.01	3.91	4.26	4.18	3.64	3.89	4.41	4.71	5.22
CKM composite or death	5.48	4.98	5.35	5.91	4.79	5.08	6.56	6.76	7.13
MACE	3.81	3.75	4.11	4.00	3.51	3.75	4.25	4.59	5.10
MACE or death	5.31	4.85	5.23	5.73	4.67	4.96	6.43	6.67	7.04
All-cause death	2.13	1.42	1.52	2.50	1.63	1.84	3.05	2.81	2.60
Safety outcomes									
Gastroparesis	0.32	0.28	0.25	0.47	0.43	0.53	0.34	0.27	0.29
Intestinal obstruction	0.56	0.51	0.46	0.65	0.64	0.70	0.63	0.64	0.61
Gallstones	0.90	0.95	0.98	1.13	1.23	1.24	0.81	0.73	0.69
Acute cholecystitis	0.23	0.24	0.26	0.31	0.34	0.31	0.21	0.15	0.16
Acute pancreatitis	0.31	0.41	0.38	0.37	0.39	0.40	0.38	0.43	0.42

Event rates are shown per 100 person-years.

Abbreviations: ITT: intention to treat; PP: per-protocol

© 2025 Derington CG et al. *JAMA Network Open*.

eTable 12. Intent-to-treat gastrointestinal adverse events among veterans initiating the study drugs.

Outcome	Unweighted: Events per 100 person years (N events/years of follow-up)			Inverse Probability Weighted: Events per 100 person years			HR (95% CI)		
	Liraglutide N = 5,527	Semaglutide N = 10,838	Dulaglutide N = 5,425	Liraglutide	Semaglutide	Dulaglutide	L vs. S	L vs. D	D vs. S
Gastroparesis	0.31 (63/20374)	0.42 (106/25170)	0.37 (71/19197)	0.32	0.47	0.34	0.68 (0.44, 1.07)	0.95 (0.64, 1.43)	0.68 (0.45, 1.03)
Intestinal obstruction	0.58 (117/20297)	0.65 (164/25093)	0.54 (104/19120)	0.56	0.65	0.63	0.87 (0.62, 1.22)	0.89 (0.60, 1.33)	0.99 (0.67, 1.45)
Gallstones	0.90 (182/20125)	1.04 (260/24965)	0.77 (146/19036)	0.90	1.13	0.81	0.81 (0.62, 1.05)	1.11 (0.83, 1.49)	0.72 (0.54, 0.95)
Acute cholecystitis	0.26 (54/20396)	0.30 (75/25226)	0.22 (42/19245)	0.23	0.31	0.21	0.76 (0.47, 1.24)	1.11 (0.69, 1.81)	0.62 (0.39, 0.99)
Acute pancreatitis	0.31 (63/20372)	0.36 (90/25199)	0.32 (61/19189)	0.31	0.37	0.38	0.85 (0.55, 1.31)	0.83 (0.48, 1.41)	1.02 (0.59, 1.76)

CI: confidence interval; D: dulaglutide; HR: hazard ratio; L: liraglutide; S: semaglutide

© 2025 Derington CG et al. *JAMA Network Open*.

eTable 13. Comparing the weighted intent-to-treat and per-protocol hazard ratios for the effectiveness outcomes among veterans initiating the study drugs.

Outcome	Liraglutide vs. semaglutide ITT	Liraglutide vs. semaglutide PP – ≥45 day	Liraglutide vs. semaglutide PP – ≥1 day	Liraglutide vs. dulaglutide ITT	Liraglutide vs. dulaglutide PP – ≥45 day	Liraglutide vs. dulaglutide PP – ≥1 day	Dulaglutide vs. semaglutide ITT	Dulaglutide vs. semaglutide PP – ≥45 day	Dulaglutide vs. semaglutide PP – ≥1 day
Gastroparesis	0.68 (0.44, 1.07)	0.55 (0.28, 1.09)	0.46 (0.25, 0.84)	0.95 (0.64, 1.43)	0.95 (0.45, 2.03)	0.85 (0.44, 1.66)	0.68 (0.45, 1.03)	0.57 (0.33, 0.99)	0.54 (0.32, 0.92)
Intestinal obstruction	0.87 (0.62, 1.22)	0.75 (0.45, 1.23)	0.63 (0.40, 0.99)	0.89 (0.60, 1.33)	0.76 (0.41, 1.44)	0.73 (0.41, 1.30)	0.99 (0.67, 1.45)	0.94 (0.56, 1.59)	0.83 (0.51, 1.35)
Gallstones	0.81 (0.62, 1.05)	0.74 (0.50, 1.11)	0.74 (0.50, 1.09)	1.11 (0.83, 1.49)	1.28 (0.80, 2.06)	1.39 (0.89, 2.15)	0.72 (0.54, 0.95)	0.58 (0.40, 0.85)	0.53 (0.37, 0.78)
Acute cholecystitis	0.76 (0.47, 1.24)	0.68 (0.31, 1.50)	0.75 (0.36, 1.58)	1.11 (0.69, 1.81)	1.64 (0.64, 4.19)	1.60 (0.71, 3.62)	0.62 (0.39, 0.99)	0.42 (0.19, 0.93)	0.49 (0.25, 0.96)
Acute pancreatitis	0.85 (0.55, 1.31)	0.97 (0.52, 1.80)	0.89 (0.51, 1.54)	0.83 (0.48, 1.41)	0.87 (0.34, 2.20)	0.88 (0.39, 2.03)	1.02 (0.59, 1.76)	1.05 (0.45, 2.41)	0.96 (0.46, 2.02)

Values are weighted HR (95% CI).

Abbreviations: CI: confidence interval; CKM: cardio-kidney metabolic; HR: hazard ratio; ITT: intention to treat; MACE: major adverse cardiovascular event; PP: per-protocol

© 2025 Derington CG et al. *JAMA Network Open*.

eFigure 1. Flowchart of veteran selection

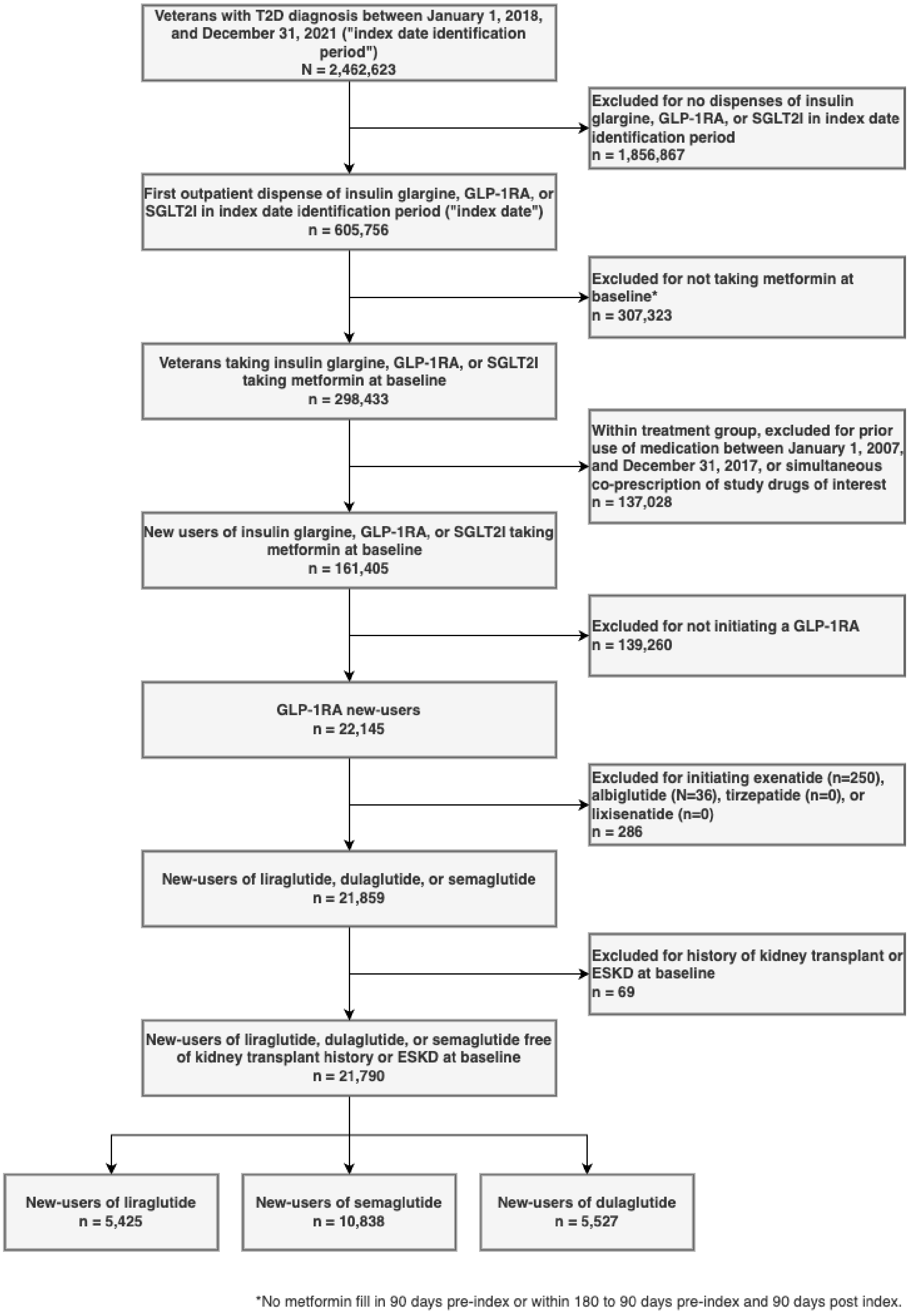

© 2025 Derington CG et al. *JAMA Network Open*.

eFigure 2. Histograms of the propensity score by treatment group.

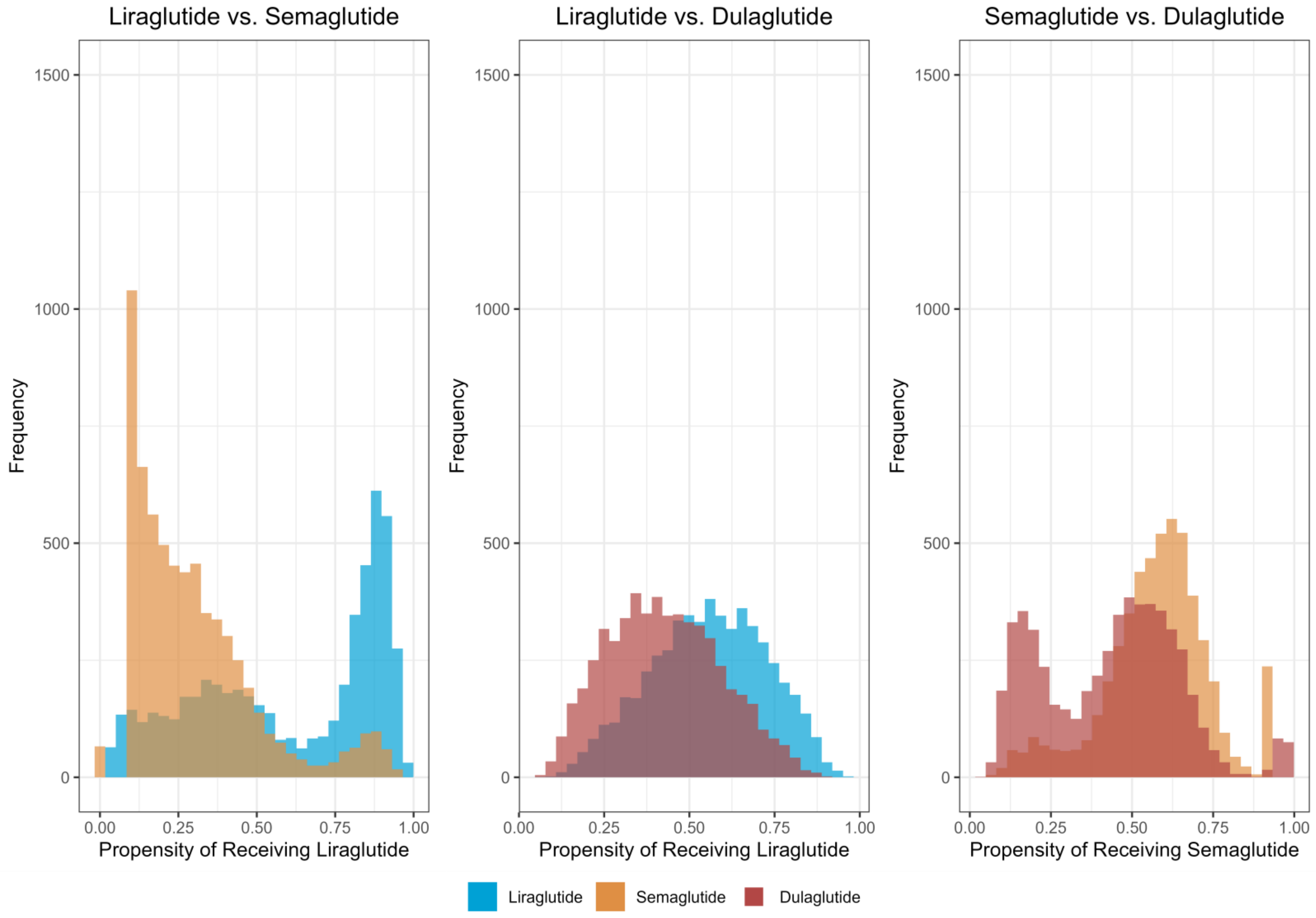

© 2025 Derington CG et al. *JAMA Network Open*.

The figure shows the distribution of propensity scores between initiators liraglutide (blue), semaglutide (yellow), and dulaglutide (red).

© 2025 Derington CG et al. *JAMA Network Open*.

eFigure 3. Balance of baseline characteristics before and after weighting.

Panel A. Maximum of three pairwise comparisons

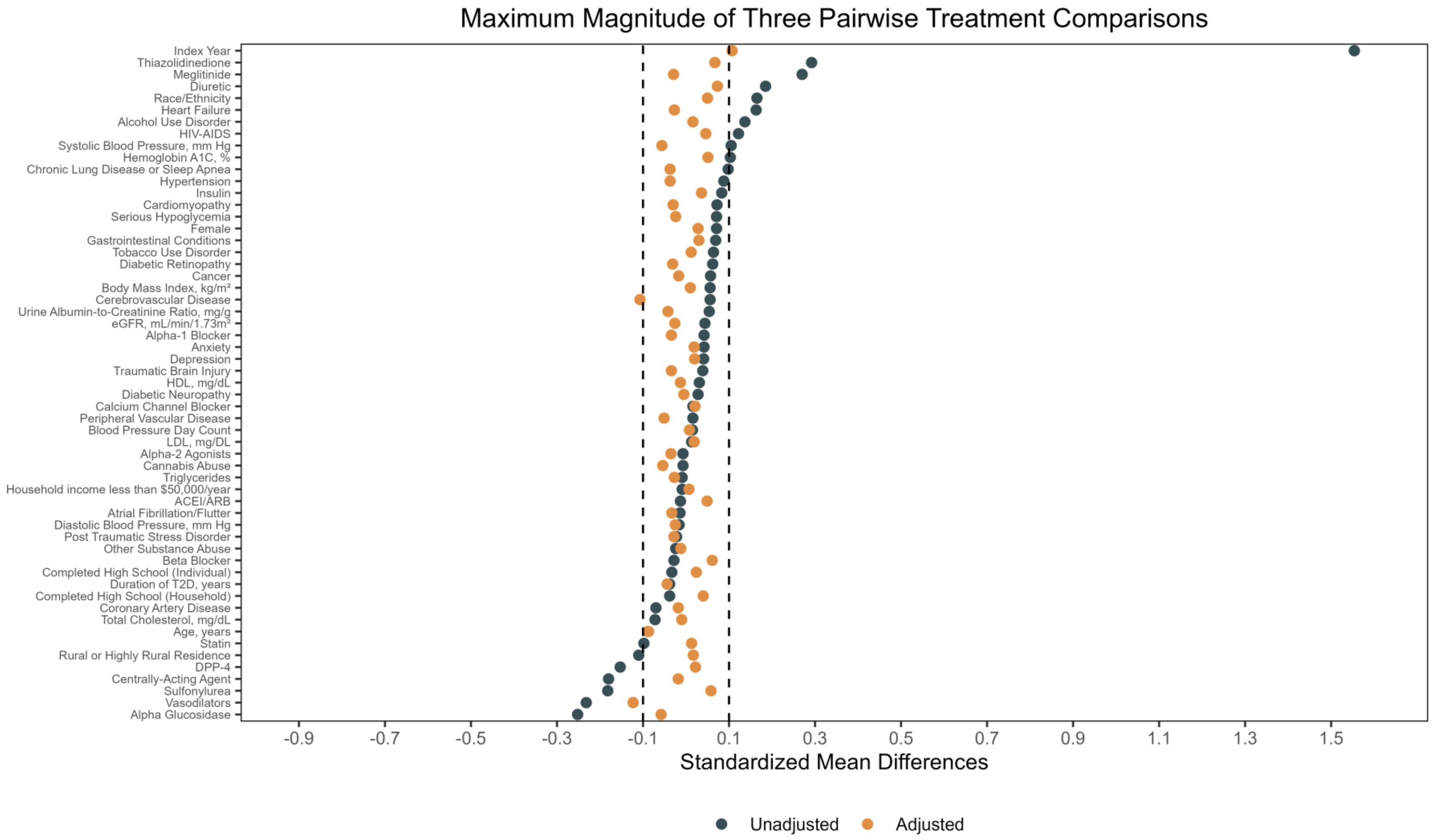

© 2025 Derington CG et al. *JAMA Network Open*.

Panel B. Liraglutide versus semaglutide

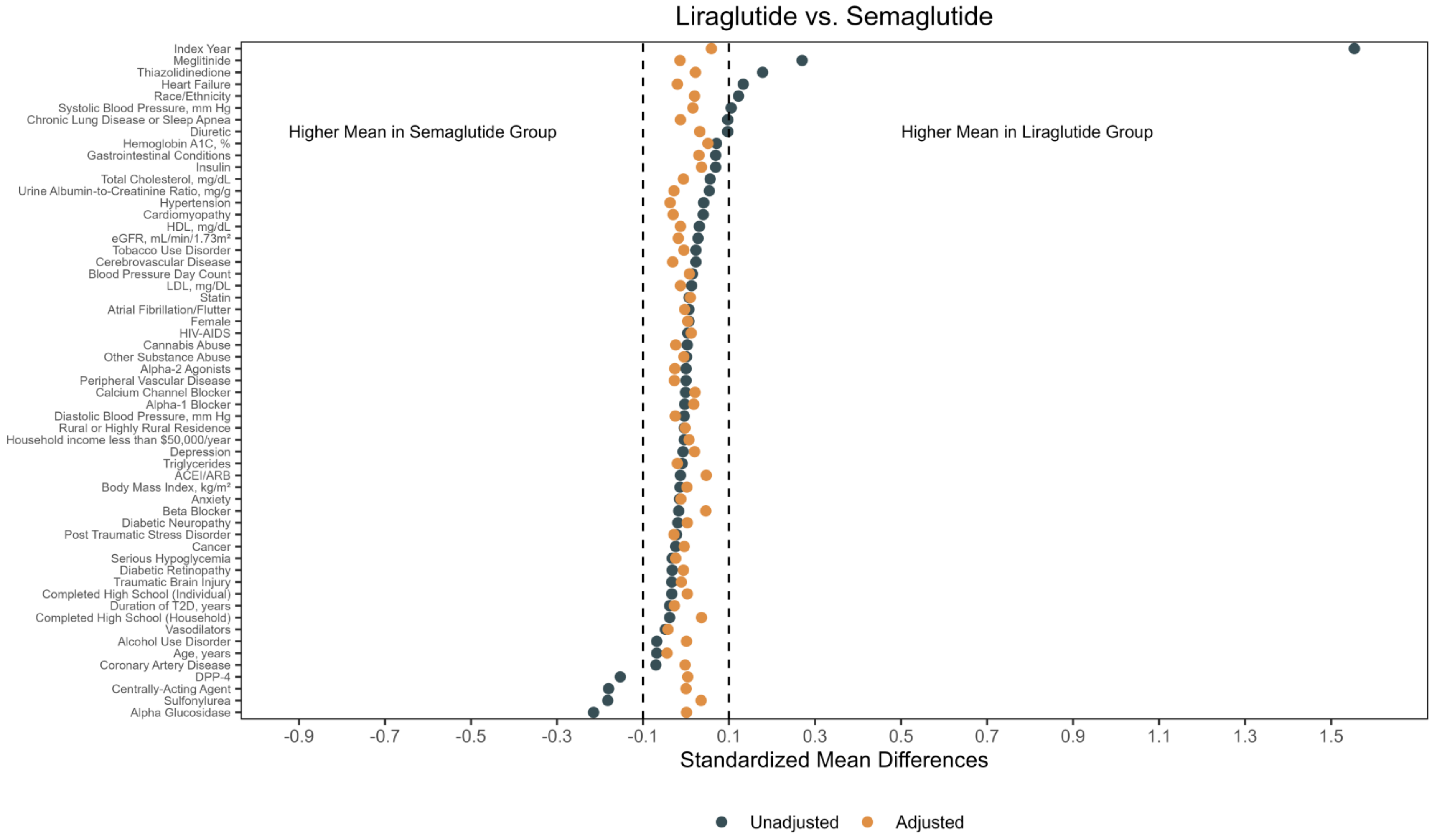

© 2025 Derington CG et al. *JAMA Network Open*.

Panel C. Liraglutide versus dulaglutide

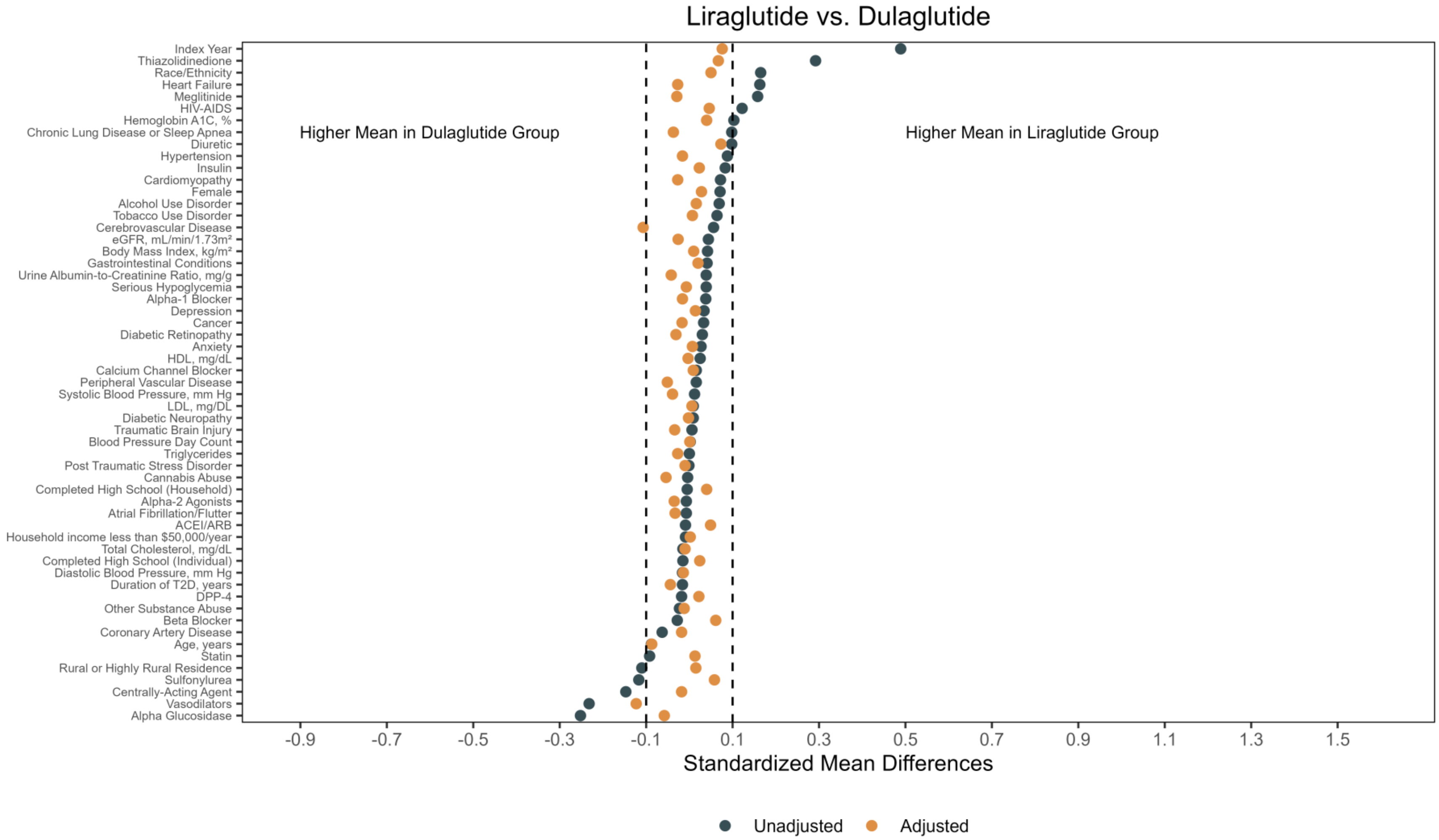

© 2025 Derington CG et al. *JAMA Network Open*.

Panel D. Dulaglutide versus semaglutide.

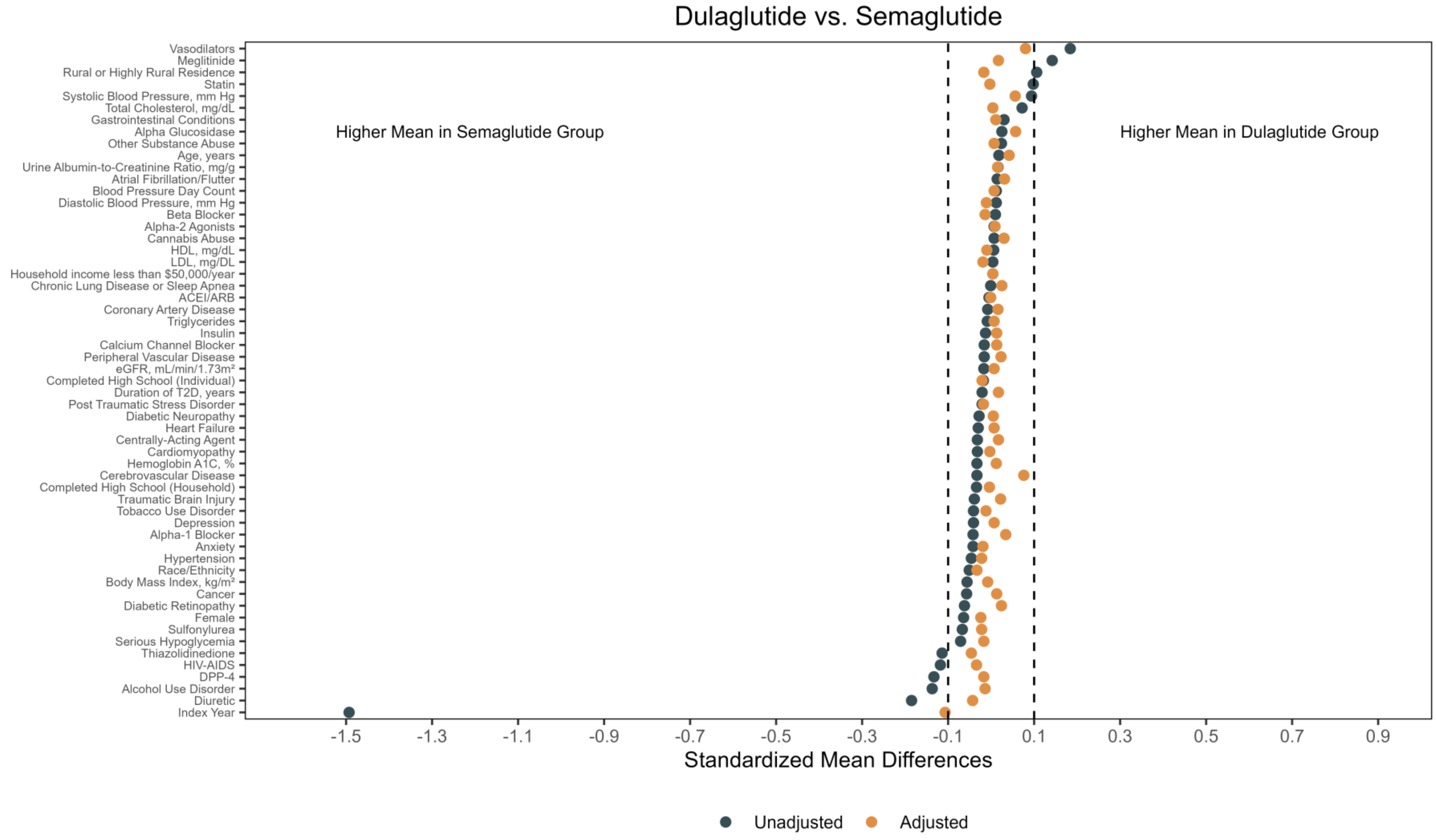

© 2025 Derington CG et al. *JAMA Network Open*.

eFigure 4: Weight change at 24 months among Veterans initiating liraglutide, semaglutide, and dulaglutide.

Panel A. Body weight change in pounds (lb)

Panel B. Body weight change from baseline (%)

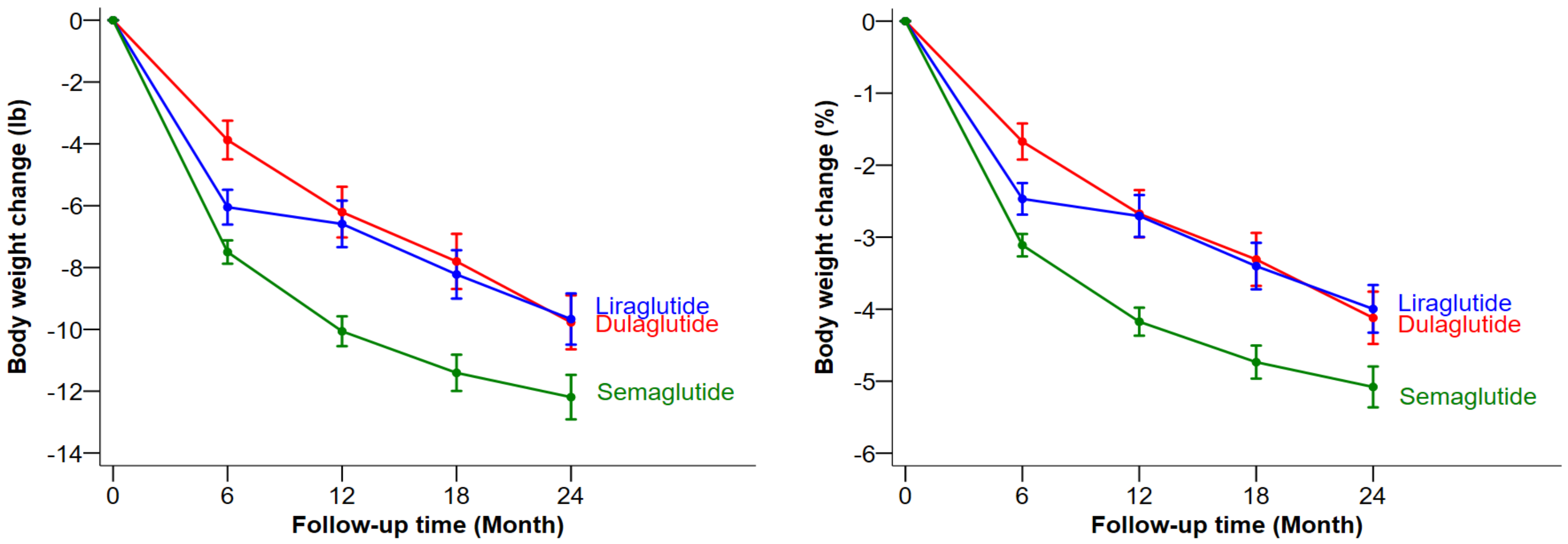

Panel A: At 24 months, p=0.86 for liraglutide vs. dulaglutide; p<.0001 for liraglutide vs. semaglutide and dulaglutide vs. semaglutide.
Panel B: At 24 months, p=0.62 for liraglutide vs. dulaglutide; p<.0001 for liraglutide vs. semaglutide and dulaglutide vs. semaglutide.
Results are adjusted; see eMethods for analytic details.

© 2025 Derington CG et al. *JAMA Network Open*.

eFigure 5: Subgroup results.

The x-axis is plotted on a logarithmic scale to appropriately display hazard ratios. *Abbreviations:* A1c: glycated hemoglobin A1c; BMI: body mass index; CI: confidence interval; CKD: chronic kidney disease; CVD: cardiovascular disease; eGFR: estimated glomerular filtration rate; T2D: type 2 diabetes; UACR: urine albumin to creatinine ratio

Panel A: Liraglutide vs. semaglutide

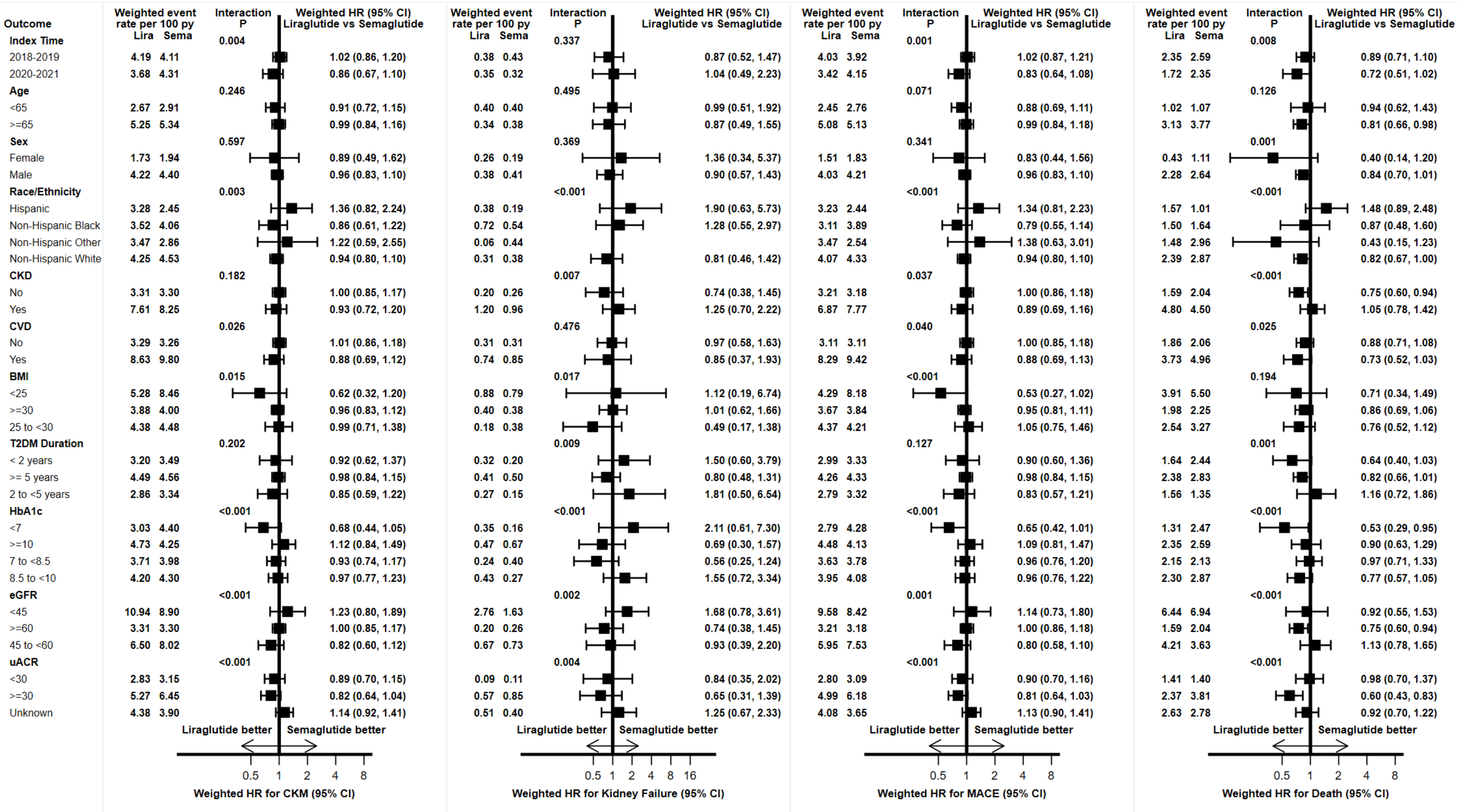

Outcome	CKM: Weighted event rate per 100 py Lira	CKM: Sema	CKM: Interaction P	CKM: Weighted HR (95% CI) Liraglutide vs Semaglutide	Kidney Failure: Weighted event rate per 100 py Lira	Kidney Failure: Sema	Kidney Failure: Interaction P	Kidney Failure: Weighted HR (95% CI) Liraglutide vs Semaglutide	MACE: Weighted event rate per 100 py Lira	MACE: Sema	MACE: Interaction P	MACE: Weighted HR (95% CI) Liraglutide vs Semaglutide	Death: Weighted event rate per 100 py Lira	Death: Sema	Death: Interaction P	Death: Weighted HR (95% CI) Liraglutide vs Semaglutide
**Index Time**			0.004				0.337				0.001				0.008	
2018-2019	4.19	4.11		1.02 (0.86, 1.20)	0.38	0.43		0.87 (0.52, 1.47)	4.03	3.92		1.02 (0.87, 1.21)	2.35	2.59		0.89 (0.71, 1.10)
2020-2021	3.68	4.31		0.86 (0.67, 1.10)	0.35	0.32		1.04 (0.49, 2.23)	3.42	4.15		0.83 (0.64, 1.08)	1.72	2.35		0.72 (0.51, 1.02)
**Age**			0.246				0.495				0.071				0.126	
<65	2.67	2.91		0.91 (0.72, 1.15)	0.40	0.40		0.99 (0.51, 1.92)	2.45	2.76		0.88 (0.69, 1.11)	1.02	1.07		0.94 (0.62, 1.43)
>=65	5.25	5.34		0.99 (0.84, 1.16)	0.34	0.38		0.87 (0.49, 1.55)	5.08	5.13		0.99 (0.84, 1.18)	3.13	3.77		0.81 (0.66, 0.98)
**Sex**			0.597				0.369				0.341				0.001	
Female	1.73	1.94		0.89 (0.49, 1.62)	0.26	0.19		1.36 (0.34, 5.37)	1.51	1.83		0.83 (0.44, 1.56)	0.43	1.11		0.40 (0.14, 1.20)
Male	4.22	4.40		0.96 (0.83, 1.10)	0.38	0.41		0.90 (0.57, 1.43)	4.03	4.21		0.96 (0.83, 1.10)	2.28	2.64		0.84 (0.70, 1.01)
**Race/Ethnicity**			0.003				<0.001				<0.001				<0.001	
Hispanic	3.28	2.45		1.36 (0.82, 2.24)	0.38	0.19		1.90 (0.63, 5.73)	3.23	2.44		1.34 (0.81, 2.23)	1.57	1.01		1.48 (0.89, 2.48)
Non-Hispanic Black	3.52	4.06		0.86 (0.61, 1.22)	0.72	0.54		1.28 (0.55, 2.97)	3.11	3.89		0.79 (0.55, 1.14)	1.50	1.64		0.87 (0.48, 1.60)
Non-Hispanic Other	3.47	2.86		1.22 (0.59, 2.55)	0.06	0.44			3.47	2.54		1.38 (0.63, 3.01)	1.48	2.96		0.43 (0.15, 1.23)
Non-Hispanic White	4.25	4.53		0.94 (0.80, 1.10)	0.31	0.38		0.81 (0.46, 1.42)	4.07	4.33		0.94 (0.80, 1.10)	2.39	2.87		0.82 (0.67, 1.00)
**CKD**			0.182				0.007				0.037				<0.001	
No	3.31	3.30		1.00 (0.85, 1.17)	0.20	0.26		0.74 (0.38, 1.45)	3.21	3.18		1.00 (0.86, 1.18)	1.59	2.04		0.75 (0.60, 0.94)
Yes	7.61	8.25		0.93 (0.72, 1.20)	1.20	0.96		1.25 (0.70, 2.22)	6.87	7.77		0.89 (0.69, 1.16)	4.80	4.50		1.05 (0.78, 1.42)
**CVD**			0.026				0.476				0.040				0.025	
No	3.29	3.26		1.01 (0.86, 1.18)	0.31	0.31		0.97 (0.58, 1.63)	3.11	3.11		1.00 (0.85, 1.18)	1.86	2.06		0.88 (0.71, 1.08)
Yes	8.63	9.80		0.88 (0.69, 1.12)	0.74	0.85		0.85 (0.37, 1.93)	8.29	9.42		0.88 (0.69, 1.13)	3.73	4.96		0.73 (0.52, 1.03)
**BMI**			0.015				0.017				<0.001				0.194	
<25	5.28	8.46		0.62 (0.32, 1.20)	0.88	0.79		1.12 (0.19, 6.74)	4.29	8.18		0.53 (0.27, 1.02)	3.91	5.50		0.71 (0.34, 1.49)
>=30	3.88	4.00		0.96 (0.83, 1.12)	0.40	0.38		1.01 (0.62, 1.66)	3.67	3.84		0.95 (0.81, 1.11)	1.98	2.25		0.86 (0.69, 1.06)
25 to <30	4.38	4.48		0.99 (0.71, 1.38)	0.18	0.38		0.49 (0.17, 1.38)	4.37	4.21		1.05 (0.75, 1.46)	2.54	3.27		0.76 (0.52, 1.12)
**T2DM Duration**			0.202				0.009				0.127				0.001	
< 2 years	3.20	3.49		0.92 (0.62, 1.37)	0.32	0.20		1.50 (0.60, 3.79)	2.99	3.33		0.90 (0.60, 1.36)	1.64	2.44		0.64 (0.40, 1.03)
>= 5 years	4.49	4.56		0.98 (0.84, 1.15)	0.41	0.50		0.80 (0.48, 1.31)	4.26	4.33		0.98 (0.84, 1.15)	2.38	2.83		0.82 (0.66, 1.01)
2 to <5 years	2.86	3.34		0.85 (0.59, 1.22)	0.27	0.15		1.81 (0.50, 6.54)	2.79	3.32		0.83 (0.57, 1.21)	1.56	1.35		1.16 (0.72, 1.86)
**HbA1c**			<0.001				<0.001				<0.001				<0.001	
<7	3.03	4.40		0.68 (0.44, 1.05)	0.35	0.16		2.11 (0.61, 7.30)	2.79	4.28		0.65 (0.42, 1.01)	1.31	2.47		0.53 (0.29, 0.95)
>=10	4.73	4.25		1.12 (0.84, 1.49)	0.47	0.67		0.69 (0.30, 1.57)	4.48	4.13		1.09 (0.81, 1.47)	2.35	2.59		0.90 (0.63, 1.29)
7 to <8.5	3.71	3.98		0.93 (0.74, 1.17)	0.24	0.40		0.56 (0.25, 1.24)	3.63	3.78		0.96 (0.76, 1.20)	2.15	2.13		0.97 (0.71, 1.33)
8.5 to <10	4.20	4.30		0.97 (0.77, 1.23)	0.43	0.27		1.55 (0.72, 3.34)	3.95	4.08		0.96 (0.76, 1.22)	2.30	2.87		0.77 (0.57, 1.05)
**eGFR**			<0.001				0.002				0.001				<0.001	
<45	10.94	8.90		1.23 (0.80, 1.89)	2.76	1.63		1.68 (0.78, 3.61)	9.58	8.42		1.14 (0.73, 1.80)	6.44	6.94		0.92 (0.55, 1.53)
>=60	3.31	3.30		1.00 (0.85, 1.17)	0.20	0.26		0.74 (0.38, 1.45)	3.21	3.18		1.00 (0.86, 1.18)	1.59	2.04		0.75 (0.60, 0.94)
45 to <60	6.50	8.02		0.82 (0.60, 1.12)	0.67	0.73		0.93 (0.39, 2.20)	5.95	7.53		0.80 (0.58, 1.10)	4.21	3.63		1.13 (0.78, 1.65)
**uACR**			<0.001				0.004				<0.001				<0.001	
<30	2.83	3.15		0.89 (0.70, 1.15)	0.09	0.11		0.84 (0.35, 2.02)	2.80	3.09		0.90 (0.70, 1.16)	1.41	1.40		0.98 (0.70, 1.37)
>=30	5.27	6.45		0.82 (0.64, 1.04)	0.57	0.85		0.65 (0.31, 1.39)	4.99	6.18		0.81 (0.64, 1.03)	2.37	3.81		0.60 (0.43, 0.83)
Unknown	4.38	3.90		1.14 (0.92, 1.41)	0.51	0.40		1.25 (0.67, 2.33)	4.08	3.65		1.13 (0.90, 1.41)	2.63	2.78		0.92 (0.70, 1.22)

© 2025 Derington CG et al. *JAMA Network Open*.

Panel B: Liraglutide vs. dulaglutide

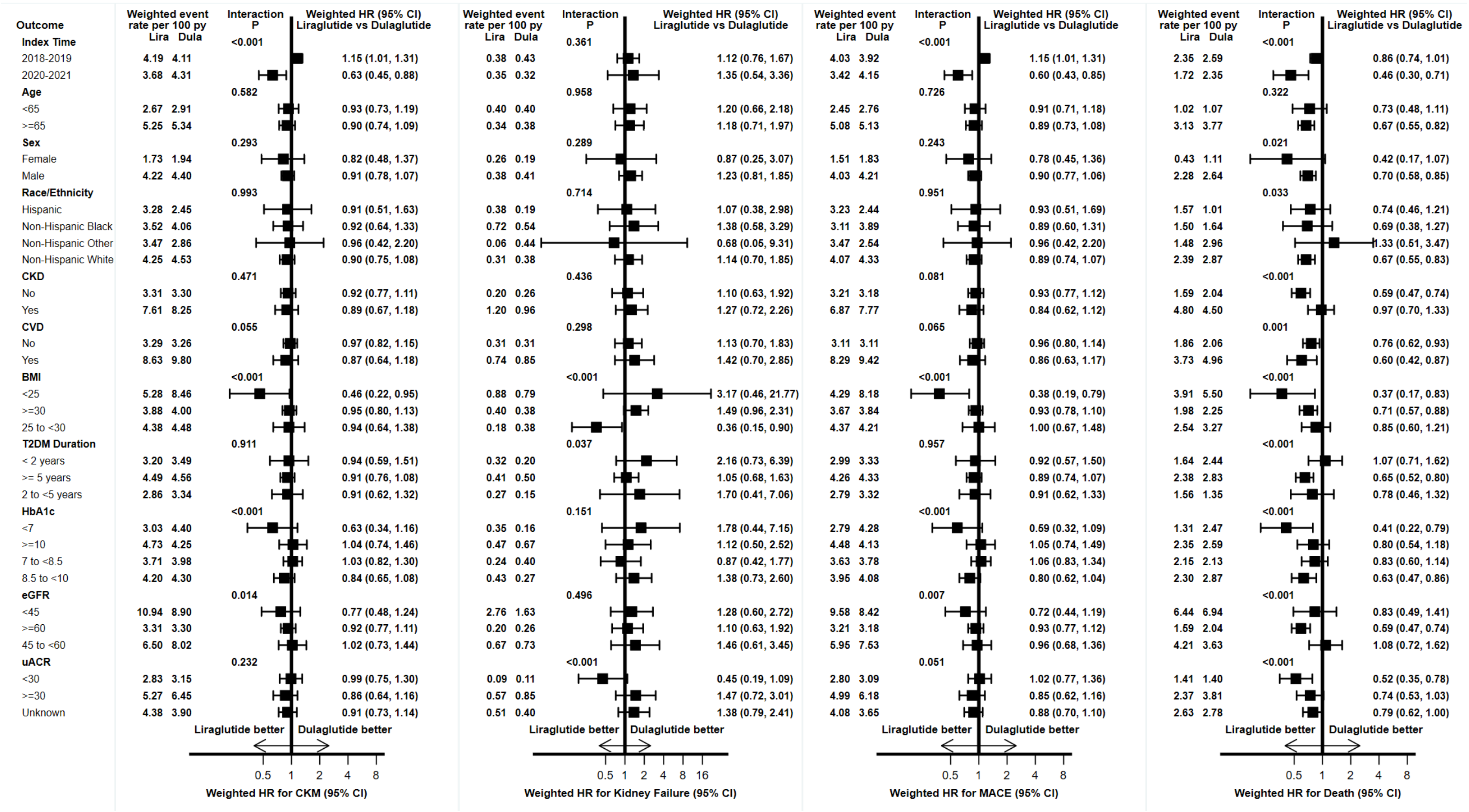

© 2025 Derington CG et al. *JAMA Network Open*.

Panel C: Dulaglutide vs. semaglutide

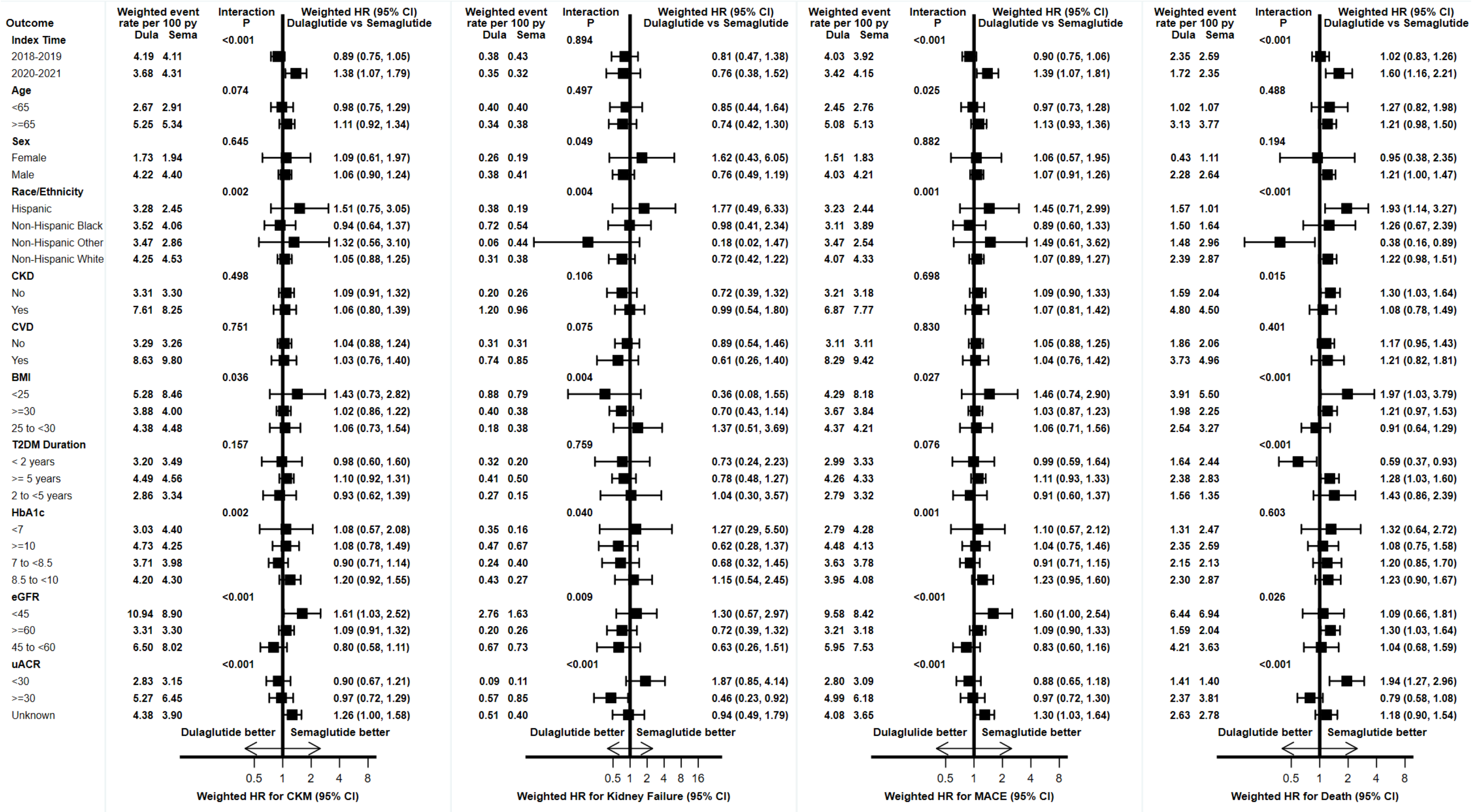

© 2025 Derington CG et al. *JAMA Network Open*.

eReferences

1. US Department of Veterans Affairs. Veterans Affairs Informatics and Computing Infrastructure (VINCI) [Internet]. [cited 2024 Jul 26];Available from: https://www.hsrd.research.va.gov/for_researchers/vinci/.
2. US Department of Veterans Affairs. VIReC Research User Guides [Internet]. [cited 2024 Jul 26];Available from: https://www.virec.research.va.gov/Resources/RUGs.asp
3. Hernán MA, Hernández-Díaz S. Beyond the intention-to-treat in comparative effectiveness research. In: Clinical Trials. 2012. p. 48–55.
4. Hernán MA, Robins JM. Per-Protocol Analyses of Pragmatic Trials. *N Engl J Med*. 2017;377:1391–1398.
5. Diggle P. Analysis of Longitudinal Data. 2nd ed. Oxford, United Kingdom: Oxford University Press; 2013.
6. Cole SR, Hernán MA, Margolick JB, Cohen MH, Robins JM. Marginal Structural Models for Estimating the Effect of Highly Active Antiretroviral Therapy Initiation on CD4 Cell Count. *Am J Epidemiol*. 2005;162:471–478.

© 2025 Derington CG et al. *JAMA Network Open*.
