# Supplementary material for: Liraglutide vs Semaglutide vs Dulaglutide in Veterans With Type 2 Diabetes
Source: JAMA Netw Open. 2025 Oct 13;8(10):e2537297. doi: 10.1001/jamanetworkopen.2025.37297 (PMC12519307; doi:10.1001/jamanetworkopen.2025.37297)
Supplement: Supplement 2. — Data Sharing Statement [file jamanetwopen-e2537297-s002.pdf]

Data Sharing Statement

Derington. Liraglutide vs Semaglutide vs Dulaglutide in Veterans With Type 2 Diabetes. *JAMA Netw Open*. Published October 13, 2025. doi:10.1001/jamanetworkopen.2025.37297

Data
Data available: No
